# Connectivity Analysis in EEG Data: A Tutorial Review of the State of the Art and Emerging Trends

**DOI:** 10.3390/bioengineering10030372

**Published:** 2023-03-17

**Authors:** Giovanni Chiarion, Laura Sparacino, Yuri Antonacci, Luca Faes, Luca Mesin

**Affiliations:** 1 Mathematical Biology and Physiology, Department Electronics and Telecommunications, Politecnico di Torino, 10129 Turin, Italy; 2 Department of Engineering, University of Palermo, 90128 Palermo, Italy

**Keywords:** EEG, functional connectivity, data-driven, signal acquisition, pre-processing, source localization

## Abstract

Understanding how different areas of the human brain communicate with each other is a crucial issue in neuroscience. The concepts of structural, functional and effective connectivity have been widely exploited to describe the human connectome, consisting of brain networks, their structural connections and functional interactions. Despite high-spatial-resolution imaging techniques such as functional magnetic resonance imaging (fMRI) being widely used to map this complex network of multiple interactions, electroencephalographic (EEG) recordings claim high temporal resolution and are thus perfectly suitable to describe either spatially distributed and temporally dynamic patterns of neural activation and connectivity. In this work, we provide a technical account and a categorization of the most-used data-driven approaches to assess brain-functional connectivity, intended as the study of the statistical dependencies between the recorded EEG signals. Different pairwise and multivariate, as well as directed and non-directed connectivity metrics are discussed with a pros–cons approach, in the time, frequency, and information-theoretic domains. The establishment of conceptual and mathematical relationships between metrics from these three frameworks, and the discussion of novel methodological approaches, will allow the reader to go deep into the problem of inferring functional connectivity in complex networks. Furthermore, emerging trends for the description of extended forms of connectivity (e.g., high-order interactions) are also discussed, along with graph-theory tools exploring the topological properties of the network of connections provided by the proposed metrics. Applications to EEG data are reviewed. In addition, the importance of source localization, and the impacts of signal acquisition and pre-processing techniques (e.g., filtering, source localization, and artifact rejection) on the connectivity estimates are recognized and discussed. By going through this review, the reader could delve deeply into the entire process of EEG pre-processing and analysis for the study of brain functional connectivity and learning, thereby exploiting novel methodologies and approaches to the problem of inferring connectivity within complex networks.

## 1. Introduction

The human brain has always fascinated researchers and neuroscientists. Its complexity lies in the combined spatial- and temporal-evolving activities that different cerebral networks explicate over three-dimensional space. These networks display distinct patterns of activity in a resting state or during task execution, but also interact with each other in various spatio-temporal modalities, being connected both by anatomical tracts and by functional associations [1]. In fact, to understand the mechanisms of perception, attention, and learning; and to manage neurological and mental diseases such as epilepsy, neurodegeneration, and depression, it is necessary to map the patterns of neural activation and connectivity that are both spatially distributed and temporally dynamic.

The analysis of the complex interactions between brain regions has been shaping the research field of connectomics [2], a neuro-scientific discipline that has become more and more renowned over the last few years [3]. The effort to map the human connectome, which consists of brain networks, their structural connections, and functional interactions [2], has given life to a number of different approaches, each with its own specifications and interpretations [4,5,6,7,8]. Some of these methods, such as covariance structural equation modeling [9] and the dynamic causal modeling [10,11], are based on the definition of an underlying structural and functional model of brain interactions. Conversely, some others, such as Granger causality [12], transfer entropy [13], directed coherence [14,15], partial directed coherence [16,17], and the directed transfer function [18], are data-driven and based on the statistical analysis of multivariate time series. Interestingly, while non-linear model-free and linear model-based approaches are apparently unrelated, as they look at different aspects of multivariate dynamics, they become clearly connected if some assumptions, such as the Gaussianity of the joint probability distribution of the variables drawn from the data [19,20], are met. Under these assumptions, connectivity measures such as Granger causality and transfer entropy, as well as coherence [21] and mutual information rate [22,23], can be mathematically related to each other. This equivalence forms the basis for a model-based frequency-specific interpretation of inherently model-free information-theoretic measures [24]. Furthermore, emerging trends, such as the development of high-order interaction measures, are coming up in the neurosciences to respond to the need for providing more exhaustive descriptions of brain-network interactions. These measures allow one to deal with multivariate representations of complex systems [25,26,27], showing their potential for disentangling physiological mechanisms involving more than two units or subsystems [28]. Additionally, more sophisticated tools, such as graph theory [29,30,31], are widely used to depict the functional structure of the brain intended as a whole complex network where neural units are highly interconnected with each other via different direct and indirect pathways.

Mapping the complexity of these interactions requires the use of high-resolution neuroimaging techniques. A number of brain mapping modalities have been used in recent decades to investigate the human connectome in different experimental conditions and physiological states [32,33,34], including functional magnetic resonance imaging (fMRI) [35,36,37,38], positron emission tomography, functional near-infrared spectroscopy, and electrophysiological methods such as electroencephalography (EEG), magnetoencephalography (MEG), and electrocorticography (ECoG) [14,39,40]. The most known technique used so far in this context is fMRI, which allows one to map the synchronized activity of spatially localized brain networks by detecting the changes in blood oxygenation and flow that occur in response to neuronal activity [41]. However, fMRI lacks in time resolution, and therefore cannot be entrusted with detecting short-living events, which can instead be investigated by EEG, a low-cost non-invasive imaging technique allowing one to study the dynamic relations between the activity of cortical brain regions and providing different information with respect to fMRI [38]. Being exploited in a wide range of clinical and research applications [30,42,43,44], EEG has allowed researchers to identify the spatio-temporal patterns of neuronal electric activity over the scalp with huge feasibility, thanks to advances in the technologies for its acquisition, such as the development of high-density EEG systems [45,46] and their combinations with other imaging modalities, robotics or neurostimulation [47,48,49,50].

On the whole, acquiring EEG signals is still a challenging task and requires tricks to face some delicate steps, such as positioning of the electrodes on the scalp or setting the more appropriate sampling frequency [40,51,52,53,54,55,56]. Furthermore, failure to properly perform the early stages of EEG pre-processing (e.g., resampling, channel re-referencing, data filtering, and artifact rejection) can decrease the signal-to-noise ratio and introduce unwanted artifacts into the data. Indeed, due to the lack of standardization of data preparation, it is crucial to pay attention to this delicate aspect of EEG analysis, as it can impact subsequent steps of the evaluation of connectivity among brain networks [6,40,57]. Moreover, it is well-recognized that the scalp EEG signals do not directly indicate the locations of the active neuronal populations in the brain [58,59]. Causality and connectivity measurements applied on the scalp EEG do not allow interpretation of the interacting brain sources, since the channel sites cannot be seen as approximations of the anatomical locations of sources, and then spurious connectivity can be detected between sensors on the scalp [60,61,62,63,64]. To overcome this issue, EEG source imaging has been widely applied over the past years to localize the anatomical sources (source space) of a given scalp measurement (sensor space) [65,66,67,68,69,70,71].

The core goal of this work is to provide a structured, though not exhaustive, description of the data-driven approaches for the study of EEG-based brain connectivity, with the aim of investigating the oscillatory interactions within and between neural networks. The strength of our study lies in the balance between the review of already published and widely accepted concepts for the investigation of functional brain connectivity and recent methodological approaches. The proposed measures are defined in the time, frequency, and information domains; and mathematical relationships between most of them are established under the assumptions of linearity and Gaussianity of the data. This would allow more widespread comprehension of the underlying mechanisms which govern the complex brain interactions in different physio-pathological states and experimental conditions. Moreover, this extensive presentation is accompanied by a review of the most common and tricky pitfalls occurring during the electrophysiological signal acquisition and pre-processing steps, followed by an attempt to match them with the influences they exert on the discussed connectivity metrics. Since some of the proposed measures and emerging trends have so far been poorly exploited in brain-connectivity analysis, we encourage the readers to start utilizing these concepts. Indeed, they may be useful to approach the problem of inferring connectivity from EEG recordings for the first time, or to cross new pathways.

Specifically, in Section 2, we discuss the different ways of approaching the problem of inferring brain connectivity, with a focus on the relations and the differences between the concepts of structural, functional, and effective connectivity. In this review, we focus on the notion of brain functional connectivity, which is grounded on the utilization of data-driven methods, either directed or non-directed [6,72]. In Section 3, we introduce the reader to the boundless world of data-driven functional connectivity estimation approaches, specifying some classifications and definitions in this field. In Section 4, we provide a more formal definition of the most commonly used data-driven functional-connectivity metrics in the context of time, frequency, and information-theoretic domains, with a specific focus on linear model-based approaches for Gaussian data. We review feasible, pairwise, and multivariate implementations of various non-directed and directed coupling techniques, and provide an overview of the applications in EEG brain connectivity. We highlight some of the technical challenges and introduce some of the other commonly utilized connectivity estimators and emerging trends in this field. In Section 5, we discuss the main features of EEG data acquisition and pre-processing, focusing on the crucial steps of resampling, channel re-referencing, data filtering, artifact rejection, and source localization. For each of these steps, the relationships with the discussed metrics are elucidated.

We warn the readers that there is no single optimum method for assessing brain connectivity. The efficiency of the method depends on the specific application and on the assumptions at the basis of the method itself. Different approaches to the study of brain connectivity may produce different results, even with reference to the same data. Hence, the selection of the most suitable technique to use for the investigation of brain connectivity is not straightforward as it may seem.

## 2. Brain Connectivity: An Overview of Key Topics

Brain connectivity aims at describing the patterns of interaction within and between different brain regions. This description relies on the key concept of functional integration [73], which describes the coordinated activation of systems of neural ensembles distributed across different cortical areas, as opposed to functional segregation, which instead refers to the activation of specialized brain regions. Brain connectivity encompasses various modalities of interaction between brain networks, including structural connectivity (SC), functional connectivity (FC), and effective connectivity (EC).

SC is perhaps the most intuitive concept of connectivity in the brain. It can be intended as a representation of the brain fiber pathways that traverse broad regions and correspond with established anatomical understanding [74]. As such, SC can be intended as a purely physical phenomenon.

On the other hand, the concept of FC was defined in [75] in terms of the statistical connections between the dynamic activity of neural units in different anatomical locations, and assessed via correlation or covariance. Some studies suggest that the repertoire of cortical functional configurations reflects the underlying anatomical connections, as the functional interactions between different brain areas are thought to vary according to the density and structure of the connecting pathways [74,76,77,78,79,80,81,82]. This leads to the assumption that investigating the anatomical structure of a network, i.e., how the neurons are linked together, is an important prerequisite for discovering its function, i.e., how neurons interact together, synchronizing their dynamic activity. Moreover, according to the original definition proposed in [75], FC does not relate to any specific direction or structure of the brain. Instead, it is purely based on the probabilities of the observed neural responses. No conclusions were made about the type of relationship between two brain regions. The only comparison is established via the presence or absence of statistical dependence.

Conversely, EC was originally defined in terms of the directional influence that one neural unit exerts over another, thereby requiring the generation of a mechanistic model of the cause–effect relationships. In a nutshell, while FC was intended as an observable phenomenon quantified through measures of statistical dependencies, such as correlation and mutual information, EC was determined to explain the observed functional dependencies based on a model of directed causal influences [75]. According to this outdated view, the key concept is that the analysis of EC can be reduced to model comparison or optimization. For instance, two models with and without a specific directed link are compared between each other to determine the existence of that link. Then, generative models of EC are tied to brain function hypotheses, and FC analysis prioritizes the differentiation of individuals based on their brain-activity measurements [75].

In the last two decades, these concepts have been widely discussed and have evolved towards various interpretations [7,72,83,84,85]. EC can be assessed either from the signals directly (i.e., data-driven EC) or based on an underlying model specifying the causal pathways given anatomical and functional knowledge (i.e., EC is a combination of both SC and FC) [83,84]. The most exploited data-driven methods based on time-series analysis include adaptations of Granger causality [12,86], transfer entropy [13], partial directed coherence [16,17], and the directed transfer function [18], and are designed to identify the directed transfer of information between two brain regions. Conversely, mechanistic models of EC focus on either (i) the determination of the model parameters that align with observed correlation patterns in a given task, such as in the case of the covariance structural equation modeling [9] and dynamic causal modeling [10], or (ii) perturbational approaches to investigate the degree of causal influence between two brain regions [87].

Interestingly, a distinction between directed and non-directed FC was proposed in [72] and then applied in [6]. On the contrary, EC is always directed and rests on a parameterised model of causal influences, usually expressed in terms of difference (discrete time) or differential (continuous time) equations [11,88,89,90]. Starting from these concepts, in this work we discuss brain FC intended as both non-directed and directed statistical dependencies between neural ensembles, estimated from data-driven methods based on time-series analysis. This view is in accordance with [6,72], where concepts of Granger causality have been associated with directed functional rather than directed effective connections.

## 3. Functional Connectivity: A Classification of Data-Driven Methods

The identification and quantification of functional brain interactions, and their clinical interpretations, are still challenging tasks, despite the literature providing a huge number of metrics for inferring connectivity from the acquired data, which is often described with a large amount of technical detail [4,5,6]. In this section, we highlight and discuss classifications and definitions proper of the data-driven approaches for inferring directed and non-directed FC.

FC can be studied in terms of coupling (non-directed FC) and causality (directed FC) [72]. Specifically, coupling refers to the existence of a statistical relationship between the time-dependent activities of the recorded signals over time. This is investigated through symmetrical connectivity measures quantifying the simultaneous occurrence of neurophysiological events that are spatially distant [75], such as correlation and mutual information. In contrast, causality refers to the presence of a time-lagged cause–effect relationship between two brain signals that occurs over time [91,92]. This is investigated through directed connectivity measures that examine the statistical causation from the data based on temporal correlations, such as Granger causality [12,93], directed coherence [14,15], partial directed coherence [16,17], and transfer entropy [13]. Coupling and causality can be assessed through a number of different approaches, depending on the physiological phenomenon under study, and thus on the utilized connectivity measure:

- Linear time series analysis methods, typically based on the autoregressive (AR) linear model representation of the interactions, which are thus referred to as model-based, or non-linear methods, typically based on probabilistic descriptions of the observed dynamics and thus referred to as model-free.
- Methods developed in the time, frequency, or information-theoretic domain, based on the features of the investigated signals one is interested in (respectively, temporal evolution, oscillatory content, and probabilistic structure);
- Methods treating the time series that represent the neuronal activity of (groups of) brain units as realizations of independent identically distributed (i.i.d.) random variables or identically distributed (i.d.) random processes, respectively, studied in terms of their zero-lag (i.e., static) or time-lagged (i.e., dynamic) correlation structure.
- Approaches that face the analysis of brain connectivity looking at pairs (pairwise analysis) or groups (multivariate analysis) of time series representative of the observed brain dynamics.

### 3.1. Model-Based vs. Model-Free Connectivity Estimators

AR model-based data-driven approaches typically assume linear interactions between signals. Specifically, in a linear framework, coupling is traditionally investigated by means of spectral coherence, partial coherence [16,21,94], correlation coefficient, and partial correlation coefficient [21]. On the other hand, different measures have been introduced for studying causal interactions, such as directed transfer function [18], directed coherence [14,15], partial directed coherence [16,17], and Granger causality [12,93]. Conversely, more general approaches, such as mutual information [75,95] and transfer entropy [13,96], can investigate non-linear dependencies between the recorded signals, starting from the definition of entropy given by Shannon [95] and based on the estimation of probability distributions of the observed data. Importantly, under the Gaussian assumption [19], model-free and model-based measures converge and can be inferred from the linear parametric representation of multivariate vector autoregressive (VAR) models [12,20,24].

Constituting the most employed metrics, linear model-based approaches are sufficient for identifying the wide range of oscillatory interactions that take place under the hypothesis of oscillatory phase coupling [6]. Linear-model-based approaches allow the frequency domain representations of multiple interactions in terms of transfer functions, partial coherence, and partial power spectrum decomposition [6,21]. This feature is extremely helpful in the study of brain signals that usually exhibit oscillatory components in well-known frequency bands, resulting from the activity of neural circuits operating as a network [97]. Moreover, linear AR models are frequently used in EEG studies because they provide improved resolution and smoother spectra, and can be applied to short segments of data [98]. Nevertheless, despite linear measures seeming to be more robust to noise and well-performing, even in non-linear cases [99], the latter should be used to capture and provide additional information on the existence of non-linear interactions that can remain hidden if a linear approach is used [99].

### 3.2. Time-Domain vs. Frequency-Domain Connectivity Estimators

It is important to distinguish between time- and frequency-domain techniques, as the latter reveal connectivity mechanisms related to the brain rhythms that operate within specific frequency bands [21,39]. While approaches such as correlation, mutual information, Granger causality, and transfer entropy are linked to a time-domain representation of the data, some others, such as coherence, directed transfer function, directed coherence, and partial directed coherence, assume that the acquired data are rich in individual rhythmic components and exploit frequency-domain representations of the investigated signals. Although this can be achieved through the application of non-parametric techniques (Fourier decomposition, wavelet analysis, Hilbert transformation after band-pass filtering [100]), the utilization of parametric AR models has collected great popularity, allowing one to evaluate brain interactions within specific spectral bands with physiological meanings [21]. Furthermore, time-frequency analysis approaches, which simultaneously extract spectral and temporal information [101], have been extensively used to study changes in EEG connectivity in the time-frequency domain [102,103,104], and in combination with deep learning approaches for the automatic detection of schizophrenia [105] and K-nearest neighbor classifiers for monitoring the depth of anesthesia during surgery [106].

Crucially, when the linear parametric formulation based on the assumption of Gaussianity [19,20] is adopted, information-theoretic measures such as the mutual information rate and the transfer entropy can be expanded in the frequency domain [12,24,93] for deriving their spectral counterparts. This does not imply that a spectral decomposition for the model-free measures is achievable in terms of probability distributions, but rather that the information-theoretic metrics can be retrieved as full-frequency integrals of spectral functions with physiological meaning [12,24,93]. This property is particularly relevant, as it establishes a straightforward link between time-, frequency-, and information-domain measures of coupling and causality, under the assumption of linearity and Gaussianity, thereby permitting one to look at the problem of inferring connectivity from different but interconnected perspectives.

## 4. Functional Connectivity Estimation Approaches

**Figure 1:**
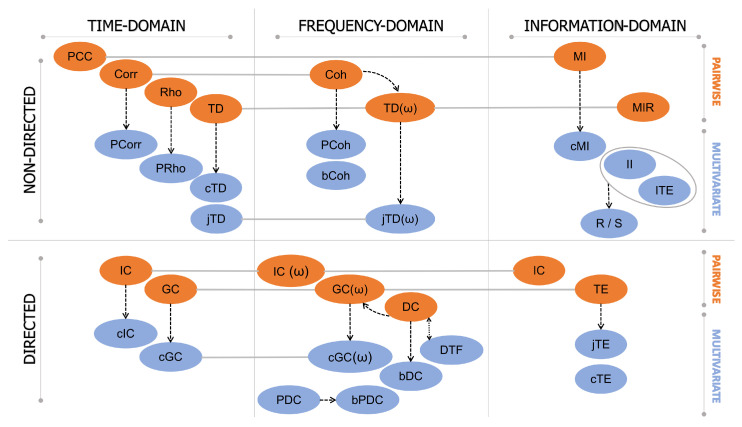
A schematic diagram of time-domain (**left**), frequency-domain (**middle**), and information-domain (**right**) measures of FC, divided into non-directed (**top** row) and directed (**bottom** row) approaches, and into pairwise (orange blocks) and multivariate (light blue blocks) methods. Continuous gray lines represent connections between domains (i.e., time, frequency, and information domain). Dashed black arrows represent connections between methods (i.e., pairwise and multivariate methods). The mathematical relation between DC and DTF is represented by a dotted bidirectional arrow. The gray ellipse surrounding the two blocks, II and ITE, reflects the equivalence of their mathematical formulation.

**Figure 2:**
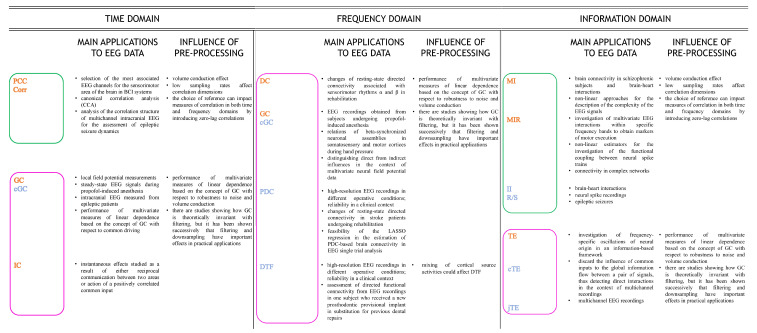
Most commonly used time-domain (**left**), frequency-domain (**middle**), and information-domain (**right**) measures of FC, divided into non-directed (green rectangles) and directed (purple rectangles) approaches, and into pairwise (orange) and multivariate (light blue) methods. For each domain, the main applications to EEG data are presented, along with the influence that pre-processing steps exert on them. References to the literature are provided for each metric, application, and impact of pre-processing.

The purpose of this section is to describe the most commonly used brain-connectivity metrics defined in the time (Section 4.1), frequency (Section 4.2), and information-theoretic domains (Section 4.3). The latter is also discussed in terms of the linear parametric formulation which is valid for Gaussian data, and mathematical connections with time- and frequency-domain measures are established. In each section, a distinction between pairwise and multivariate measures, and between directed and non-directed approaches, is made (Figure 1). In Figure 2, the brain-connectivity metrics mostly used in the revised literature are schematized, with references to their application to EEG data and the influences exerted on them by the pre-processing steps described in Section 5. Then, in Section 4.4, other common methods to infer FC from brain data, not directly associated with the classification conceived in this work, are described, and emerging trends in this field are briefly discussed. Finally, Section 4.5 is devoted to a brief discussion about the most-used approaches to assess the statistical significance for estimates of the metrics proposed in the previous sections.

### 4.1. Time-Domain Approaches

Several time-domain approaches devoted to the study of FC have been developed throughout the years. Despite phase-synchronization measures, such as the phase locking value [107] and other model-free approaches [108] being still abundantly used in brain-connectivity analysis, linear methods are easier to use and sufficient to capture brain interactions taking place under the hypothesis that neuronal interactions are governed by oscillatory phase coupling [6].

In a linear framework, ergodicity, Gaussianity, and wide-sense stationarity (WSS) conditions are typically assumed for the acquired data, meaning that the analyzed signals are stochastic processes with Gaussian properties and preserve their statistical properties as a function of time. These assumptions are made, often implicitly, as prerequisites for the analysis, in order to assure that the linear description is exhaustive and the measures can be safely computed from a single realization of the analyzed process. Under these assumptions, the dynamic interactions between a realization of *M* Gaussian stochastic processes (e.g., *M* EEG signals recorded at different electrodes) can be studied in terms of time-lagged correlations. In the time domain, the analysis is performed via a linear parametric approach grounded on the classical vector AR (VAR) model description of a discrete-time, zero-mean, stationary multivariate stochastic Markov process, $S={\left[X_{1}\ldots X_{M}\right]}^{⊺}$. Considering the time step *n* as the current time, the dynamics of S can be completely described by the VAR model [21,24]:

(1) $$S_{n}=\sum_{k=1}^{p}A_{k}S_{n-k}+U_{n},$$

where $S_{n}={\left[X_{1,n}\ldots X_{M,n}\right]}^{⊺}$ is the vector describing the present state of S, and $S_{n}^{p}={\left[S_{n-1}^{⊺}\ldots S_{n-p}^{⊺}\right]}^{⊺}$ describes its past states until lag *p*, which is the model order defining the maximum lag used to quantify interactions; $A_{k}$ is the M×M coefficient matrix quantifying the time-lagged interactions within and between the *M* processes at lag *k*; and U is a M×1 vector of uncorrelated white noise with an M×M covariance matrix $\Sigma =diag(\sigma_{11}^{2},\ldots \sigma_{MM}^{2})$. Multivariate methods based on VAR models as in (1) depend on the reliability of the fitted model, and especially the model order. While lower model orders can provide inadequate representations of the signal, orders higher than are strictly needed tend to provide overrepresentation of the oscillatory content of the process and drastically increase noise [109]. One should pay attention to the procedure for selecting the optimum model order, which can be set according to different criteria, such as the Akaike information criterion (AIC) [110] or the Bayesian information criterion (BIC) [111].

It should be noted that, in multichannel recordings such as with EEG data, the analysis can be multivariate, which means taking all the channels into account and fitting a full VAR model, as in (1), or it can be done by considering each channel pair separately, which means fitting a bivariate AR model (2AR) in the form of (1) with M=2:

(2) $$Z_{n}=\sum_{k=1}^{p}B_{k}Z_{n-k}+W_{n},$$

where $Z={\left[X_{i}X_{j}\right]}^{⊺}$, i,j=1,…,M (i≠j), is the bivariate process containing the investigated channel pair, with $Z_{n}={\left[X_{i,n}X_{j,n}\right]}^{⊺}$ and $Z_{n}^{p}={\left[Z_{n-1}^{⊺}\ldots Z_{n-p}^{⊺}\right]}^{⊺}$ describing, respectively, the present and *p* past states of Z; $B_{k}$ is the 2×2 coefficient matrix quantifying the time-lagged interactions within and between the two processes at lag *k*, and W is a 2×1 vector of uncorrelated white noises with 2×2 covariance matrix Λ. The pairwise (bivariate) approach typically provides more stable results, since it involves the fitting of fewer parameters but leads to loss of information due to the fact that only a pair of time series is taken into account [112]. Indeed, since there are various situations that provide significant estimates of connectivity in the absence of true interactions (e.g., cascade interactions or common inputs) [21,112], the core issue becomes whether the estimate of pairwise connectivity reflects a true direct connection between the two investigated signals or is the result of spurious dynamics between multiple time series. To answer this question, it is recommended to take into account the information from all channels when estimating the interaction terms between any pair of time series. Even if at the expense of increased model complexity resulting in a more difficult model identification process, moving from a pairwise to a multivariate approach can significantly increase the accuracy of the reconstructed connectivity patterns. This would allow distinguishing direct from indirect interactions through the use of extended formulations obtained through partialization or conditioning procedures [16,21,113].

#### 4.1.1. Non-Directed Connectivity Measures

Non-directed coupling relations between time series refer to associations which do not specify the direction of influence. This type of coupling does not assume causality between the time series, but rather looks for symmetrical statistical dependencies between them [21,114].

##### Pairwise Measures

In the time domain, the simplest method to find symmetrical statistical dependencies between signals is finding the correlation.

Following a so-called "static” approach, one can discard temporal correlations between the processes and study only the zero-lag correlation between $X_{i,n}$ and $X_{j,n}$, implicitly considering the processes as formed by i.i.d. random variables. This correlation can be investigated by means of the Pearson correlation coefficient $\rho_{i,j}$ (PCC), defined as the ratio between the covariance of the two variables and the product of their standard deviations. Varying in the range [−1;1], it reflects the strength and direction of the linear relationship between the two variables, but not the slope of that relationship, nor non-linear aspects [115].

In a dynamic context, by considering the processes $X_{i}$ and $X_{j}$ as formed by i.d. random variables, the pairwise cross-correlation $r_{ij,k}$ at lag *k* between $X_{i,n}$ and $X_{j,n-k}$ can be computed as the ${\left(i-j\right)}^{th}$ element of the time-lagged correlation matrix $R_{k}=E\left[Z_{n}Z_{n-k}^{⊺}\right]$ (Corr) [21]. Being a normalized version of the pairwise cross-correlation and corresponding to the dynamic version of PCC, the squared correlation coefficient (Rho) quantifies non-directed coupling in the time-domain [21,116]. It can be computed from (2) relating the covariance matrix of $Z_{n}$, $\Sigma_{Z}$, to the covariance of $W_{n}$, $\Lambda =diag(\lambda_{ii}^{2},\lambda_{jj}^{2})$, and can be intended globally or individually [117]. In the former case, it quantifies the linear interdependence between $Z_{n}$ and $Z_{n}^{p}$, and can be computed as $\rho_{Z_{n},Z_{n}^{p}}^{2}=1-\frac{\left|\Lambda \right|}{|\Sigma_{Z}|}$, where |·| is the matrix determinant. In the latter case, it quantifies the linear interdependence between $X_{i,n}$ and $Z_{n}^{p}$, and can be computed as $\rho_{X_{i,n},Z_{n}^{p}}^{2}=1-\frac{\lambda_{ii}^{2}}{\sigma_{i}^{2}}$, where $\sigma_{i}^{2}$ is the first diagonal element of $\Sigma_{Z}$, corresponding to the variance of $X_{i}$ (the same applies to $X_{j}$).

The non-directed coupling between pairs of time series can also be investigated in the time domain following a “dynamic” approach, whereby the time-lagged interactions between the two analyzed processes are investigated as a whole. This is achieved using the so-called total dependence (TD), a logarithmic measure defined by Geweke [12] which can be computed in terms of predictability improvement. To do this, the two processes $X_{i}$ and $X_{j}$ are described first by a full model in the form of (2), and then by two reduced AR models including only the past samples from the processes $X_{i}$ and $X_{j}$ taken individually—denoting the error variances of the reduced models as ${\tilde{\lambda }}_{ii}^{2}$ and ${\tilde{\lambda }}_{jj}^{2}$—the TD measure is given by [24]

(3) $$F_{i,j}=\ln\left(\frac{{\tilde{\lambda }}_{ii}^{2}{\tilde{\lambda }}_{jj}^{2}}{\left|\Lambda \right|}\right).$$

TD compares the innovations of the full and reduced AR models in a symmetric way, thereby quantifying how much the bivariate dynamic description of the two processes improves predictability compared with the individual dynamic descriptions taken separately.

##### Multivariate Measures

In a multivariate context, different combinations of the *M* processes can be considered when studying their static or dynamic correlations. For instance, one can investigate the interdependencies between a target process $X_{i}$ and two source processes, grouped in the vector $\left[X_{j}X_{z}\right]$, which can be in turn multivariate when each source comprises two or more processes. Additionally, when the analysis of connectivity is focused on the study of the interactions between blocks of time series, one can group the signals describing the activity of a certain brain area and investigate the interactions between two blocks of processes. Otherwise, the multiple interactions between the *M* processes of the network can be described through the utilization of more sophisticated tools, such as those derived from the frameworks of high-order interactions [25,26,27] or graph theory [31].

Regardless of the combination of variables chosen, the importance of using multivariate approaches stems from the observation that a correlation between pairs of investigated signals can arise when there is direct connectivity between them, but also in the presence of signals acting as sources of correlation which are not modeled. These signals represent confounders which give rise to spurious connectivity when the interest is in analyzing the interaction patterns [112], but are also crucial for the emergence of high-order interactions when the interest is in investigating complex collective behaviors [118]. In either case, the cross-correlation and the correlation coefficient, and the total dependence, are not sufficient to disambiguate these phenomena.

In particular, pairwise measures of connectivity are not helpful in assessing the presence of direct pathways of interactions, i.e., links of genuine association, between two processes [21,114]. In order to distinguish between these situations (i.e., to disambiguate direct vs. indirect coupling), the concept of partialization (or conditioning) was introduced and exploited in neuroscience [16,21,94]. In a multivariate context, the definition of partial correlation coefficient (PRho) follows the removal of the effects of all the M−2 processes different than the two processes of interest, $X_{j}$ and $X_{i}$. This is achieved via the so-called partialization procedure, which basically consists of the inversion of the correlation matrix $R_{k}$ yielding the matrix $R_{k}^{-1}$ denoted as partial correlation (PCorr) [21]. The procedure can be defined in terms of predictability improvement by comparing the innovation variances of a restricted model, whereby the target series $X_{i}$ is described as a linear combination of (M−2)×p past states of the whole network and the driver $X_{j}$, and a full model, whereby the *p* past states of $X_{j}$ are added to the regressors of the restricted model.

PRho is a measure of direct coupling, in the sense that it quantifies the linear interdependence between the processes under scrutiny after removing spurious effects due to other processes within the network. Remarkably, this partialized measure is symmetric in $X_{j}$ and $X_{i}$, exactly as is the correlation coefficient, thereby allowing one to reverse the roles of driver and target arbitrarily.

In addition to PRho, a measure of conditional TD (cTD) was also introduced [93] to discard the effects of spurious connectivity patterns between $X_{i}$ and $X_{j}$ due to a third input (either a scalar or a vector process). Computation of cTD is also based on a predictability improvement and a model comparison approach [93]. Moreover, a time-domain measure of the total coupling between two source processes taken together and the target process, i.e., between $\left[X_{j},X_{z}\right]$ and $X_{i}$, was defined in [119] as $F_{[jz];i}$. In the following, we refer to this measure as joint TD (jTD).

#### 4.1.2. Directed Connectivity Measures

The principle of causality is fundamental in time-series analysis to identify driver–response (i.e., time-lagged) relations between the processes. In this work, this principle is explored with reference to the concept of Granger causality (GC), which has been one of the most relevant approaches exploited by modern time-series analysis. The concept of GC was originally developed by Wiener [120] and then made operative by Granger in the context of linear regression models [86]. In particular, GC relates the presence of a cause–effect relation to two aspects: the cause must precede the effect in time and must carry unique information about the present value of the effect. This relationship is not symmetrical and can be bidirectional, thereby enabling the detection of directed and reciprocal influences. Differently from non-directed measures, causality approaches exploiting this concept allow focusing on specific directional pathways of interactions within the investigated network [121].

##### Pairwise Measures

The first and most common practical implementation of GC is based on linear AR modeling of two time series [86] performed under the WSS assumption, representing the result of a model comparison and thus based on the concept of predictability improvement. Specifically, the target time series $X_{i}$ is described both by a reduced AR model (including only the *p* past samples from the process $X_{i}$ itself) and by a full AR on both series (including also the *p* past samples of the driver $X_{j}$). In the time domain, the GC from driver $X_{j}$ to target $X_{i}$ is then defined as the natural logarithm of a variance ratio, where the individual variance terms reflect the residuals of the reduced (${\tilde{\lambda }}_{ii}^{2}$) and full ($\lambda_{ii}^{2}$) models fitted to the time series [86,92,113]:

(4) $$F_{j\to i}=\ln\left(\frac{{\tilde{\lambda }}_{ii}^{2}}{\lambda_{ii}^{2}}\right).$$

According to this definition, the measure of GC from $X_{i}$ to $X_{j}$ takes strictly positive values, establishing the presence of a directed link, when the past of $X_{j}$ improves the prediction of the present of $X_{i}$ above and beyond the predictability brought about already by the past of $X_{i}$ itself.

The GC measure has a close connection with the measure of TD between two processes previously defined. Indeed, if two processes interact with each other exclusively via time-lagged effects, the TD $F_{i,j}$ is exactly the sum of the GC measured along the two directions of interaction ($F_{i\to j}$ and $F_{j\to i}$) [12]. This establishes an important relation between directed and non-directed measures of connectivity. Such a relation is completed in the more general case when the processes interact also at lag zero, introducing the so-called measure of instantaneous causality $F_{i\cdot j}$ (IC) [12,24]. This measure represents the part of $F_{i,j}$ that cannot be captured by time-lagged interactions quantified via $F_{i\to j}$ and $F_{j\to i}$. In fact, the measures of TD, GC, and IC are connected by the well-known time-domain Geweke formulation [12]:

(5) $$F_{i,j}=F_{i\to j}+F_{j\to i}+F_{i\cdot j}.$$

It is worth noting that the IC is always zero in the absence of zero-lag effects between the time series, i.e., when the AR model is strictly causal [122], but this is generally not true in practical analysis, when significant fast effects occur and are no more negligible [123]. A measure of the so-called “extended GC”, quantifying both time-lagged and instantaneous effects between time series, has also been proposed [124].

##### Multivariate Measures

GC analysis is context-dependent, meaning that adding or removing processes from the VAR representation of S affects the final results. In particular, if the computation does not include all processes that have a causal link to $X_{j}$ and $X_{i}$, observing significant GC from $X_{j}$ to $X_{i}$ may be the result of hidden common drivers or intermediate processes. Unobserved latent variables might severely affect GC analysis and increase the likelihood of spurious detections of causality [112]. Therefore, conditional GC (cGC), also called partial GC, has been defined [20,93,113] to state whether the interaction between $X_{i}$ and $X_{j}$ is direct or mediated by other processes in the network S of interacting processes. In such cases, GC from $X_{j}$ to $X_{i}$ conditioned on the set of M−2 processes, represented by V=S\Z, can be computed as $F_{j\to i|v}=\ln\left(\frac{{\tilde{\sigma }}_{ii}^{2}}{\sigma_{ii}^{2}}\right)$, where ${\tilde{\sigma }}_{ii}^{2}$ is the innovation variance of a restricted model whereby the target $X_{i}$ is described from its past and the past of V, and $\sigma_{ii}^{2}$ is the innovation variance of the full model whereby the *p* past lags of the driver $X_{j}$ are added to the regressors.

Conditional IC (cIC) has also been defined in the context of an AR parametric representation of the data [93]. The implementation of cIC follows the same rationale as cGC, based on predictability improvement and model comparison. It is worth noting that the Geweke decomposition in (5) can be extended to the case of conditional measures, thereby allowing one to define cTD as the sum of the two cGC terms and the cIC term [93].

#### 4.1.3. Applications of Time-Domain Approaches to EEG Data

A huge number of applications to EEG data of the time-domain measures defined above are found in the literature.

The investigation of correlation patterns through PCC, despite it being a widely accepted measure of the statistical relationships between signals [115], is rarely found in applications to brain data. To get information on brain connectivity with and without consideration of the volume-conduction effect, the complex PCC was defined as a unique single measure to provide information on phase locking and weighted phase lag [125]. Later on, the imaginary component of the complex PCC was proposed to investigate the effects of photobiomodulation on brain connectivity in an elderly person with probable memory and thinking disorder [126]. The PCC technique was also recently employed to select the most associated EEG channels for the sensorimotor area of the brain, in brain–computer interface (BCI) systems [127]. Canonical correlation analysis (CCA), defined by [128] and exploiting the PCC method, was used for classification of evoked or event-related potentials in EEG data [129]. An extension of CCA, called group-sparse CCA, was proposed for simultaneous EEG channel selection and emotion recognition [130]. Other applications of CCA include the assessment of mental stress effects on prefrontal cortical activities using a combined fNIRS-EEG approach [131].

Regarding dynamic correlations, epileptic seizure dynamics were assessed in [132] by analyzing the correlation structure of multichannel intracranial EEG, whereas in [133], the influence of the choice of reference on linear multivariate EEG correlation patterns was investigated, along with the effect of static (i.e., zero-lag) correlations on brain connectivity.

Causality measures based on GC time-domain formulation have been extensively used in EEG analysis, although active discussion does exist on their reliability in recovering the functional structure of brain networks [134,135,136,137,138]. Despite a wide range of implementations including non-linear [139,140,141], non-parametric [142], and adaptive [143,144] modeling of the cause–effect relations in brain connectivity, predictability-improvement methods have been utilized for inferring GC among EEG signals from multivariate realizations. Thanks to high temporal resolution, data obtained from EEG recordings of continuous neural activity are well suited to GC analysis [91]. The Wiener–Granger concept of causality has been used with local-field potential measurements from the cat visual system [145,146] and with steady-state EEG signals during propofol-induced anesthesia [147] to evaluate the directionality of cortical interactions. Predictability-improvement GC indexes have been used on intracranial EEG measured from epileptic patients, documenting the difficulty of properly assessing pairwise GC in the presence of several interacting processes and stressing the need for fully multivariate approaches [121]. Multivariate measures of linear dependence based on the concept of GC, and their performances with respect to robustness to noise, volume conduction, and common driving, are discussed in [148].

Still, an important aspect to be investigated is the presence of zero-lag interactions among brain signals. Specifically in EEG analysis, instantaneous effects may be often encountered as a result of either reciprocal communication between two areas (bidirectional interaction) or action of a positively correlated common input with no significant relative time delay, or even a combination of both [149,150,151]. In [149], the Geweke formulation (5) [12] was applied to study the contributions of the two above factors to near-zero phase-lag while exploiting local field potentials. Negative correlation was found between the phase-lag and instantaneous causality, implying that the stronger the common input, the closer to zero the phase lag. The study suggests that instantaneous causality should be accounted for when dealing with highly interconnected neural data, since disregarding it may have a great impact on the computation of GC indexes [152].

### 4.2. Frequency-Domain Approaches

To examine oscillatory neuronal interactions and identify the individual rhythmic components in the measured data, representations of connectivity in the frequency domain are often desirable. The transformation from the time domain to the frequency domain can be carried out by exploiting parametric (model-based) or non-parametric (model-free) approaches. Non-parametric signal-representation techniques are mostly based on the definition of the power spectral density (PSD) matrix of the process as the Fourier transform (FT) of $R_{k}$, on the wavelet transformation (WT) of data, or on Hilbert transformation following band-pass filtering [100]. In general, they bypass the issues of the ability of linear AR models to correctly interpret neurophysiological data and the selection of the optimum model order. The latter choice can be problematic, especially with brain data, because it strongly depends on the experimental task, the quality of the data and the model estimation technique [153]. However, the non-parametric spectral approach is somewhat less robust compared to parametric estimates, since it can be characterized by lower spectral resolution; e.g., it has been shown to be less efficient in discriminating epileptic occurrences in EEG data [154].

On the other hand, parametric approaches exploit the frequency-domain representation of the VAR model, in the multivariate (1) or in the bivariate (2) case, which means computing the model coefficients in the Z-domain and then evaluating the model transfer function H(ω) on the unit circle of the complex plane, where $\omega =2\pi \frac{f}{f_{s}}$ is the normalized angular frequency and $f_{s}$ is the sampling frequency [21]. The M×M PSD matrix can then be computed using spectral factorization as

(6) $$P\left(\omega \right)=H\left(\omega \right)\Sigma H^{*}\left(\omega \right),$$

where * stands for the Hermitian transpose [21]; note that Σ is replaced by Λ in the case of (2), i.e., when M=2. It is worth noting that, while the frequency-domain descriptions ubiquitously used and reviewed here are based on the VAR model representation, their key element is actually the spectral factorization theorem reported above and that approaches other than VAR models can be used to derive frequency-domain connectivity measures [155].

In the following, we show the derivation of spectral measures of brain connectivity considering the traditional parametric spectral analysis of the process Z (or S in the multivariate case).

#### 4.2.1. Non-Directed Connectivity Measures

##### Pairwise Measures

The frequency-domain counterpart of the time-domain pairwise cross-correlation is the spectral coherence. From the spectral analysis of the process Z, the elements of the obtained 2×2 PSD matrix P(ω) are combined to define the so-called complex coherence (Coh) function $\Gamma_{ij}\left(\omega \right)$:

(7) $$\Gamma_{ij}\left(\omega \right)=\frac{P_{ij}\left(\omega \right)}{\sqrt{P_{ii}\left(\omega \right)P_{jj}\left(\omega \right)}},$$

where $P_{ij}\left(\omega \right)$ is the cross-spectral density (CSD) between $X_{i}$ and $X_{j}$ estimated at frequency ω, and $P_{ii}\left(\omega \right)$ is the PSD of $X_{i}$ (the same applies to $P_{jj}\left(\omega \right)$). The squared modulus of Coh is generally used to quantify the frequency-specific linear relationship between $X_{i}$ and $X_{j}$, ranging between 0 (no dependence) and 1 (maximal dependence) [21]. Noteworthily, as happens for time-domain correlation measures, this measure is symmetric in $X_{i}$ and $X_{j}$.

The squared Coh can be represented on a logarithmic scale through the spectral measure of TD [12]:

(8) $$f_{i,j}\left(\omega \right)=\ln\left(\frac{P_{ii}\left(\omega \right)P_{jj}\left(\omega \right)}{\left|P(\omega )\right|}\right)=-\ln\left(1-|\Gamma_{ij}\left(\omega \right){|}^{2}\right).$$

Geweke derived this spectral measure requiring the fulfillment of some properties (which here we refer to as “requirements of Geweke”). First, the measure has to be non-negative; moreover, it has to fulfill the so-called spectral integration property, since it can be shown that $F_{i,j}=\frac{1}{2\pi }\int_{-\pi }^{\pi }f_{i,j}\left(\omega \right)d\omega$ [12,24]. This property establishes a link between the time- and frequency-domain representations of the total dependence between two processes, which are extended to the information domain in the next section.

##### Multivariate Measures

As for the PRho in the time domain, the definition of the squared partial coherence (PCoh) follows the removal of the effects of all remaining M−2 processes but $X_{j}$ and $X_{i}$ [16,21,94], according to the partialization procedure consisting of the inversion of the PSD matrix. PCoh is a measure of direct coupling, quantifying the linear frequency-specific interdependence between the two time series after removing spurious effects due to other processes within the network. This measure is symmetric in $X_{j}$ and $X_{i}$ exactly as the squared Coh.

While PCoh is still computed considering two scalar processes $X_{i}$ and $X_{j}$ and conditioning on a third vector variable including all the remaining processes of the considered network, it is worth mentioning that an alternative approach does exist when dealing with multivariate datasets. Indeed, Coh can also be computed between two multivariate processes, each with a given dimensionality, which in turn can model time-series data from two blocks of channels (or regions of interest), thereby yielding the so-called block coherence (bCoh) [156]. This acquires importance, especially when one wants to assess the non-directed relationships between two brain networks. Each network is characterized by a set of recording channels.

Interestingly, a frequency-domain measure of the total coupling between two source processes taken together and the target process, i.e., between $\left[X_{j},X_{z}\right]$ and $X_{i}$, was defined in [119] as $f_{[jz];i}\left(\omega \right)$. Its time-domain counterpart, i.e., the jTD, can be retrieved by taking its integral, i.e., $F_{[jz];i}=\frac{1}{2\pi }\int_{-\pi }^{\pi }f_{[jz];i}\left(\omega \right)d\omega$ [119].

#### 4.2.2. Directed Connectivity Measures

##### Pairwise Measures

The strength of the frequency-specific causal interactions from $X_{j}$ to $X_{i}$ can be evaluated via power-spectrum decomposition [21,157] through the squared modulus of the directed coherence (DC) [14,15], computed as

(9) $$|\gamma_{ij}{\left(\omega \right)|}^{2}=\frac{\lambda_{jj}^{2}{\left|H_{ij}\left(\omega \right)\right|}^{2}}{P_{ii}\left(\omega \right)},$$

where $|H_{ij}{\left(\omega \right)|}^{2}$ is the element of the 2×2 transfer function matrix H(ω) describing the lagged influence of $X_{j}$ on $X_{i}$. This quantity was derived independently in [158], where it was denoted as causal coherence. It represents the causal contribution of $X_{j}$ to $X_{i}$ under the hypothesis of strict causality [21,159]. Importantly, an extended version of DC (eDC) was introduced [160] by computing DC on a 2AR model including instantaneous effects from one process to another in the form of model coefficients, i.e., by allowing the lag *k* to take the zero value as well.

An extension of the concept of pairwise causality to the frequency-domain representation of time series was formulated earlier by Geweke [12] and later widely discussed in terms of application to neuroscience data [113] and in the context of information theory [24]. The computation of pairwise GC indexes as a function of frequency is based on: (i) fitting the observed set of time series with a linear parametric model as in (2); (ii) representing the model coefficients in the Fourier domain; (iii) deriving the frequency-dependent causal relations among signals starting from the definition of DC in (9). Indeed, it is possible to show that, under the assumption of strict causality, there exists a relationship between the frequency-specific GC $f_{j\to i}\left(\omega \right)$ and the DC in (9), the former being defined as the logarithmic counterpart of the latter [24,122]:

(10) $$f_{j\to i}\left(\omega \right)=\ln\left(\frac{P_{ii}\left(\omega \right)}{\lambda_{ii}^{2}{\left|H_{ii}\left(\omega \right)\right|}^{2}}\right)=-\ln\left(1-|\gamma_{ij}\left(\omega \right){|}^{2}\right).$$

Interestingly, $f_{j\to i}\left(\omega \right)$ fulfills the spectral integration property; i.e., its integration over the whole frequency axis provides the time-domain estimate of GC: $F_{j\to i}=\frac{1}{2\pi }\int_{-\pi }^{\pi }f_{j\to i}\left(\omega \right)d\omega$ [12,24]. The same definitions hold for $f_{i\to j}\left(\omega \right)$. Furthermore, the spectral measure of IC $f_{i\cdot j}\left(\omega \right)$ was defined ad hoc to satisfy a spectral decomposition similar to (5) [12,24]:

(11) $$f_{i,j}\left(\omega \right)=f_{i\to j}\left(\omega \right)+f_{j\to i}\left(\omega \right)+f_{i\cdot j}\left(\omega \right).$$

Contrary to the other terms of (11) and to its time-domain counterpart, the spectral IC does not fulfill the requirements of Geweke. Indeed, it may be negative for some frequencies and has no clear physical meaning [24], resulting in non-zero estimates at frequency ω, even in the case of strict causality [122].

##### Multivariate Measures

In a multivariate context, the DC and the spectral GC can be defined again as in (9) and (10), respectively, where the transfer function elements $H_{ij}\left(\omega \right)$ and $H_{ii}\left(\omega \right)$ are taken from the M×M transfer matrix, which also includes information relevant to all M−2 processes besides $X_{i}$ and $X_{j}$. The DC and the spectral GC between two processes modeled within a fully multivariate setting are sensitive to both direct influences and indirect actions mediated by other signals, and as such, they do not constitute frequency-domain measures of causality, intended in the Granger sense, for multivariate processes.

Granger causality can be computed in the frequency domain through the so-called partial directed coherence $\pi_{ij}\left(\omega \right)$ (PDC), evaluating the direct pathway from $X_{j}$ to $X_{i}$ not mediated by other processes within the network [16,17]. The ability to infer directed effects is granted to the PDC by the fact that it is formulated from the spectral representation of the VAR model coefficients instead of the transfer function; this advantage is balanced by the fact that using the coefficients hinders interpretability of the modulus of the PDC compared to the DC [21]. Squared versions of PDC in its different normalizations are usually adopted, due to higher stability and accuracy [161,162]. Many variants of the PDC estimator have been recently provided. The generalized version of PDC (gPDC) introduced by [163] shares with the Coh, PCoh, and DC functions the desirable property of scale-invariance, contrary to the original PDC, which may be affected by differences in the amplitudes of the considered signals. Later on, an extended version (ePDC) was introduced [160] by computing PDC of a VAR model, including instantaneous effects from one process to another in the form of model coefficients. Additionally, the information PDC (iPDC) was introduced [164] to provide a precise interpretation of PDC in terms of the mutual information between partialized processes, establishing it as a measure of direct connectivity strength (it reduces to the PDC when Σ equals the identity matrix). The equivalence of all these measures in terms of the connectivity pattern they provide was demonstrated in [165].

It is worth mentioning that DC and PDC can also be computed between two multivariate processes, as happens for Coh, yielding the so-called block DC (bDC) and block PDC (bPDC), respectively [114]. This acquires importance, especially when one wants to assess the directed (block DC) and the partial and directed (block PDC) relationships between two brain networks of recording channels.

In order to determine the directional influences between the components in a multivariate system, the directed transfer function (DTF) was proposed [18]: it is a normalized version of the model transfer function describing the ratio of the influence of $X_{j}$ on $X_{i}$ to all the influences on $X_{i}$. It was first interpreted as a measure of causality in a multivariate sense [166], but this reading was confuted in [167], where the DTF was associated with a spectral measure quantifying the total causal influence from one component to another, thereby not being strictly related to multivariate or pairwise GC.

It should be underlined that, both for a bivariate process, as in (2), and for a fully multivariate process, the DTF is equivalent to the concept of DC in (9) when input variances are all equal [168]. As for the iPDC, the information DTF (iDTF) was introduced [164] to provide an interpretation of DTF in terms of the mutual information between partialized processes (it reduces to the DTF is case Σ equals the identity matrix).

In a multivariate framework consisting of three processes, $X_{i}$, $X_{j}$, and $X_{z}$, the concept of direct causality between $X_{i}$ and $X_{j}$ can be investigated by means of spectral cGC $f_{j\to i|z}\left(\omega \right)$, defined in [93] and derived from an appropriately normalized moving-average representation for $X_{i}$ and $\left[X_{j}X_{z}\right]$. This measure fulfills the spectral integration property as the spectral GC in (10), thereby allowing one to retrieve the time-domain cGC $F_{j\to i|z}$ as its integral over frequencies.

#### 4.2.3. Applications of Frequency-Domain Approaches to EEG Data

The usefulness of DC, DTF, and PDC has been demonstrated in neuroscience. DTF and PDC were applied to high-resolution EEG recordings in different operative conditions and suggested their reliability in a clinical context [39]. The normalized DTF was used for assessing directed functional connectivity from EEG recordings in one subject who received a new prosthodontic provisional implant as a substitute for previous dental repairs [169]. DC and gPDC were exploited to investigate the changes in resting-state directed connectivity associated with sensorimotor rhythms α and β, occurring in stroke patients who followed a rehabilitation treatment [170]. The results suggest that using different methods to measure directed connectivity can improve the understanding of how brain motor regions are connected between each other. The least absolute shrinkage and selection operator (LASSO) regression was used in the estimation of PDC-based brain connectivity when few data samples were available, as in EEG single trial analysis [171]. PDC was used to quantify directed interactions on the scalp under resting-state conditions in stroke patients undergoing a rehabilitation treatment based on BCIs [68]. The statistical properties of PDC were discussed, and the method was applied to EEG data in a subject suffering from essential tremor [172]. In [173], an algorithm based on directed connectivity between brain sites was developed, and the measure of gPDC was used to analyze interictal periods from long-term iEEG signals.

All of the above-mentioned analyses were performed under the assumption of stationary EEG data. However, as non-stationarity in EEG dynamics has often been pointed out in the literature, estimators such as Coh and PDC have been developed with a time-variant approach, using adaptive AR or state-space models [174,175].

Furthermore, frequency-domain GC approaches have also been often exploited in brain-connectivity analysis. Spectral GC was applied to EEG recordings obtained from subjects undergoing propofol-induced anesthesia [147]. GC was exploited to investigate the relations of beta-synchronized neuronal assemblies in somatosensory and motor cortices during hand pressure as a part of a visual discrimination task for monkeys [150]. Extended formulations of GC, including conditional (or partial) GC [176,177,178,179], have been widely used to deal with brain networks made up by several nodes. The potential of conditional GC compared to the traditional pairwise GC in distinguishing direct from indirect influences was clearly shown in the context of multivariate neural-field potential data [176].

### 4.3. Information-Domain Approaches

The statistical dependencies among electrophysiological signals can be evaluated using information theory. Concepts of mutual information, mutual information rate, and information transfer are widely used to assess the information exchanged between two interdependent systems [75,95], the dynamic interdependence between two systems per unit of time [22,23], and the dynamic information transferred to the target from the other connected systems [13,96], respectively. The main advantage of these approaches lies in the fact that they are probabilistic and can thus be stated in a fully model-free formulation. On the other hand, their practical assessment in the information domain is not straightforward because it comprises the estimation of high-dimensional probability distributions [180], which becomes more and more difficult as the number of network nodes increases in multichannel EEG recordings. Nevertheless, information-based metrics can also be expressed in terms of predictability improvement, such that their computation can rely on linear parametric AR models, where concepts of prediction error and conditional entropy, GC and information transfer, or TD and mutual information rate, have been linked to each other [19,20,121,181]. Indeed, it has been demonstrated that, under the hypothesis of Gaussianity, predictability improvement and information-based indexes are equivalent [19]. Based on the knowledge that stationary Gaussian processes are fully described in terms of linear regression models, a central result is that for Gaussian data, all the information measures can be computed straightforwardly from the variances of the innovation processes of full and restricted AR models [19]. These equivalences, which establish the links between information-theoretic and time-domain measures of connectivity, are explored in the next subsections.

#### 4.3.1. Non-Directed Connectivity Measures

##### Pairwise Measures

Non-directed information-based measures quantify static (mutual information) or dynamic (mutual information rate) symmetric interrelationships between two random variables or stochastic processes, respectively.

The mutual information (MI) quantifies the information shared between two random variables based on the concept of Shannon entropy [23,95]. In the analysis of two stationary random processes, $X_{i}$ and $X_{j}$, the MI is dependent on the time lag separating the two variables taken from the processes. A common choice is to compute the MI at lag zero, i.e., between the variables $X_{i,n}$ and $X_{j,n}$:

(12) $$I\left(X_{i,n};X_{j,n}\right)=H\left(X_{i,n}\right)+H\left(X_{j,n}\right)-H\left(X_{i,n},X_{j,n}\right),$$

where H(·) is the entropy of a single variable, measuring the amount of information carried by the variable, and H(·,·) is the joint entropy of two variables, quantifying information as the average uncertainty of their states taken together. In the linear parametric framework, the MI between $X_{i,n}$ and $X_{j,n}$ has a logarithmic relation with the squared PCC [75,117], i.e., $I\left(X_{i,n};X_{j,n}\right)=-\frac{1}{2}\ln\left(1-\rho_{i,j}^{2}\right)$.

The MI rate (MIR) is an extension of MI to the case in which the whole processes $X_{i}$ and $X_{j}$ are considered in place of the random variables sampling them. The MIR is a dynamic measure whereby the time-lagged interactions between the processes are quantified [22,23]:

(13) $$I_{X_{i};X_{j}}=H_{X_{i}}+H_{X_{j}}-H_{X_{i},X_{j}},$$

where $H_{X_{i}}$ is the entropy rate of $X_{i}$, quantifying the density of the average information in the process, formulated as the conditional entropy of the present of the process given its past [182] (the same holds for $H_{X_{j}}$), and $H_{X_{i},X_{j}}$ is the entropy rate of $X_{i}$ and $X_{j}$ taken together.

It is worth noting that, in a linear model-based framework, when the processes have a joint Gaussian distribution, the MIR can be represented in the frequency domain as the logarithmic counterpart of the spectral coherence [22,183]:

(14) $$i_{X_{i};X_{j}}\left(\omega \right)=-\frac{1}{2}\ln\left(1-|\Gamma_{i,j}\left(\omega \right){|}^{2}\right);$$

this relates the spectral MIR to the frequency-domain TD measure given in (8), and consequently allows one to retrieve the MIR in the time domain as $I_{X_{i};X_{j}}=\frac{1}{2}F_{i,j}$ thanks to the spectral integration property [24]. The latter relation establishes a close link between the MIR and the TD measure for pairs of Gaussian processes. Noteworthily, in [183] it was shown that the frequency-specific measures of bPDC/bDC and canonical PDC/DC (cPDC/cDC), the latter retrieved employing canonical decomposition to reveal the main frequency-domain modes of interaction between the two blocks, have information-theoretic interpretations in terms of MIR.

##### Multivariate Measures

Extensions of the MI to the multivariate case have also been developed. The conditional MI $I(X_{i,n},X_{j,n}|X_{z,n})$ (cMI) between two variables taken synchronously from the processes $X_{i}$ and $X_{j}$, given a third variable taken from a third process, $X_{z}$, was defined to quantify the residual MI between $X_{i,n}$ and $X_{j,n}$ when $X_{z,n}$ is known [95,184]. A measure of multivariate MI between blocks of interacting series grouped in two vector variables was defined in [117], where the global correlation coefficient was considered in place of the pairwise PCC.

Another interesting multivariate information-theoretic quantity derived from the concept of MI is the interaction information (II), which quantifies the concept of information modification and is used in neuroscience to study how different signals interact with each other to convey information to a target signal [185,186,187,188,189,190,191,192]. Specifically, II measures the amount of information that a target variable shares with two sources, which can also be vector variables, when they are taken individually but not when they are taken together [185,186,193]. As for the MI in (12), in the analysis of two stationary random processes $X_{i}$ and $X_{j}$, the II is computed at lag zero, i.e., between the target $X_{i,n}$ and the source variables $X_{j,n}$ and $X_{z,n}$:

(15) $$I\left(X_{i,n};X_{j,n};X_{z,n}\right)=I\left(X_{i,n};X_{j,n}\right)+I\left(X_{i,n};X_{z,n}\right)-I\left(X_{i,n};X_{j,n},X_{z,n}\right).$$

Noteworthily, contrary to other information measures such as the MI and the MIR, the II can take on both positive and negative values. Positive values indicate redundancy (i.e., $I\left(X_{i,n};X_{j,n},X_{z,n}\right)<I\left(X_{i,n};X_{j,n}\right)+I\left(X_{i,n};X_{z,n}\right)$), and negative values indicate synergy (i.e., $I\left(X_{i,n};X_{j,n},X_{z,n}\right)>I\left(X_{i,n};X_{j,n}\right)+I\left(X_{i,n};X_{z,n}\right)$) between the two interacting sources sending information to the target. The concept of II can be extended to the case in which the whole processes, $X_{i}$ and $X_{j}$, are considered in place of the random variables sampling them, whereby $I_{X_{i};X_{j};X_{z}}$ quantifies the dynamic information shared between the present of the target, its past, and the pasts of both sources [193]. It is worth noting that, in a linear model-based framework, this concept of “dynamic” II, can be linked to the time- and frequency-domain formulations of TD (3) and jTD:

(16) $$I_{X_{i};X_{j};X_{z}}=F_{i,j}+F_{i,z}-F_{jz,i};$$

(17) $$i_{X_{i};X_{j};X_{z}}\left(\omega \right)=f_{i,j}\left(\omega \right)+f_{i,z}\left(\omega \right)-f_{jz,i}\left(\omega \right).$$

The two measures satisfy the properties of II for random variables [185,186] in time (16) and frequency (17) domains. The property of spectral integration, i.e., $I_{X_{i};X_{j};X_{z}}$, can be retrieved as the integral of $i_{X_{i};X_{j};X_{z}}\left(\omega \right)$, along with the properties of redundancy and synergy, which hold for each specific frequency in case of (17).

Redundancy (R) and synergy (S) are two key concepts in the field of information theory. Redundancy refers to two sources conveying the same information to the target, and synergy refers to two sources interacting independently in the transmission of information to the target. Different definitions and computational approaches for these concepts were proposed during the last decade [185,186,187,188,189,190,191,192]. Specifically, the so-called partial information decomposition (PID) was proposed in [187] to separately quantify redundancy and synergy as positive quantities, according to an expansion of the overall interaction between the target and the two sources that includes “unique” information contributions of each source to the target. These definitions were developed in the framework of causality, i.e., considering directed interactions from the sources taken individually or together to the target, and they are discussed in the next subsection. Nonetheless, in the case of non-directed interplay between the considered variables, a frequency-specific decomposition of the spectral jTD $f_{jz,i}\left(\omega \right)$ was defined in [119] following the philosophy of PID, where non-directed redundancy and synergy were computed and quantified unequivocally assuming redundancy as the minimum of the interaction between each individual source and the target [188,194]. According to this approach, it is possible to rewrite (17) as $i_{X_{i};X_{j};X_{z}}\left(\omega \right)=r_{\left[X_{j}X_{z}\right];X_{i}}\left(\omega \right)-s_{\left[X_{j}X_{z}\right];X_{i}}\left(\omega \right)$, where *r* and *s* refer to redundancy and synergy, respectively. Importantly, $i_{X_{i};X_{j};X_{z}}\left(\omega \right)$ quantifies the “net” redundancy, which is intended as the balance between the redundant and synergistic contributions the sources share with the target. The frequency-specific PID measures can be integrated to yield equivalent information-rate measures.

#### 4.3.2. Directed Connectivity Measures

##### Pairwise Measures

Information transfer is a key component of information processing. It can be measured by a variety of directed information measures, of which transfer entropy (TE) is the most popular. Pairwise TE from the driver $X_{j}$ to the target $X_{i}$ plays a central role in the evaluation of information-based causality indexes. It quantifies how much $X_{j}$ influences $X_{i}$ by comparing the probability of finding the target in a present state $X_{i,n}$ given its *p* past states $X_{i,n}^{p}$. The probability of the same state includes the *p* past states of the driver $X_{j,n}^{p}$ [13,96]:

(18) $$T_{j\to i}=I\left(X_{i,n};X_{j,n}^{p}|X_{i,n}^{p}\right)=H\left(X_{i,n}|X_{i,n}^{p}\right)-H\left(X_{i,n}|X_{i,n}^{p},X_{j,n}^{p}\right).$$

While this definition of causality is expressed in terms of probability distributions, information-domain causality can also be expressed in terms of predictability improvement by exploiting linear parametric models and assuming Gaussianity [19,121,181]. If this is the case, TE quantifies how much the prediction of the current state of the target from its own past improves when the past of the driver is added to the linear model. In this framework, it has been demonstrated that twice, the TE equals the parametric time-domain GC in (4)—i.e., $T_{j\to i}=\frac{1}{2}F_{j\to i}$[19]. It is worth noting that, as $F_{j\to i}$ owns a spectral representation in terms of DC as in (10), an extension of information-domain pairwise causality to the frequency domain is available for Gaussian data, with $T_{j\to i}=\frac{1}{4\pi }\int_{-\pi }^{\pi }f_{j\to i}\left(\omega \right)d\omega$ [24,27].

An information-theoretic symmetric measure quantifying the instantaneous information shared between $X_{i}$ and $X_{j}$ can be defined as $I_{i\cdot j}=I\left(X_{i,n};X_{j,n}|X_{i,n}^{p},X_{j,n}^{p}\right)$, which is computed after removing the common information with the past states of the processes [24,195]. This can also be interpreted in a linear framework and obtained as the integral of the spectral IC, i.e., of the quantity $\frac{1}{2}f_{i\cdot j}\left(\omega \right)$. Consequently, the Geweke decompositions in time (5) and frequency domains (11) can be extended to the information theory, thereby allowing one to decompose the MIR (13), (14) as

(19) $$I_{X_{i};X_{j}}=T_{i\to j}+T_{j\to i}+I_{i\cdot j}.$$

##### Multivariate Measures

Extensions of TE to the multivariate analysis of time series have been proposed to account for the effects of third inputs on the investigated interaction pathways.

According to the so-called information transfer decomposition (ITD) [196], which can be thought of as an alternative to the PID, two multivariate quantities of directed coupling between pairs of processes can be determined.

The joint TE (jTE) [196] has been defined as a metric quantifying the overall (direct and indirect) causal information flow transferred to a putative target process $X_{i}$ from the past of all the other sources considered together. In the case of three variables, where $X_{i}$ is the target process and $\left[X_{j}X_{z}\right]$ is the source vector, jTE can be computed as:

(20) $$T_{jz\to i}=T_{j\to i}+T_{z\to i}-I_{j;z|i}^{i}$$

where $I_{j;z|i}^{i}$ is the so-called interaction transfer entropy (ITE) between $X_{j}$ and $X_{z}$ to $X_{i}$, quantifying the interaction information transfer measured as the dynamic II of the present of the target and the past of the two sources conditioned to the past of the target (i.e., $I(X_{i,n};X_{j,n}^{p};X_{z,n}^{p}|X_{i,n}^{p})$) [193].

The decomposition of jTE in (20) is not unique, since it has been demonstrated that jTE can be written in terms of partial TE (or conditional TE, cTE) as $T_{jz\to i}=T_{j\to i|z}+T_{z\to i|j}-I_{j;z|i}^{i}$, where $T_{j\to i|z}$ and $T_{z\to i|j}$ are cTEs quantifying the information transfer from one source to the target conditioned to the other source. cTE allows one to discard the influence of a third input to the global information flow between a pair of signals.

The concepts of non-directed redundancy and synergy discussed above can be extended to the case of directed interactions between the source vector $\left[X_{j}X_{z}\right]$ and the target $X_{i}$. In such a case, the PID can be applied to retrieve the jTE as [194]:

(21) $$T_{jz\to i}=U_{j\to i}+U_{z\to i}+R_{jz\to i}+S_{jz\to i},$$

where $U_{j\to i}=T_{j\to i}-R_{jz\to i}$ and $U_{z\to i}=T_{z\to i}-R_{jz\to i}$ are the unique TEs measuring the unique information transferred from each individual source to the target; and $R_{jz\to i}$ and $S_{jz\to i}$ are the redundant and synergistic TEs quantifying the redundant and synergistic information transferred from the two sources to the target, respectively. Interestingly, while a frequency-specific representation has been implemented [119] for the non-directed measures of the PID decomposition, the same does not hold for the directed measures above (i.e., $U_{j\to i}$, $U_{z\to i}$, $R_{jz\to i}$, and $S_{jz\to i}$). However, further development in this field can occur, which started with the work of Faes et al. [27], whereby concepts of information theory and frequency domain were linked, together exploiting a linear parametric formulation of stochastic processes in multivariate networks based on state-space modeling.

#### 4.3.3. Applications of Information-Domain Approaches to EEG Data

The use of information-theoretic analysis is growing in popularity within the field of neuroscience, particularly for evaluating brain connectivity through quantification of the information transfer between brain nodes. This is particularly relevant when using GC as a measure of information transfer due to its connection with TE, thereby allowing for a straightforward linear parametric formulation of the information-theoretic quantities [19].

Information-based directed connectivity measures are among the most widely utilized in brain-connectivity analysis. TE has been widely used to detect patterns of functional directed connectivity in brain networks, especially by exploiting model-free approaches [197,198,199]. Linear estimators have also been used, where the TE has been associated with the concept of spectral GC [27,181,200], thereby opening the possibility to investigate frequency-specific oscillations of neural origin in an information-based framework. Moreover, cTE has allowed researchers to discard the influences of common inputs to the global information flow between a pair of signals, thereby detecting direct interactions in the context of multichannel recordings [196,201]. jTE has been successfully used in multichannel EEG recordings [194].

MI has been successfully used in neuroscience to investigate connectivity in brain networks, e.g., in schizophrenic subjects [202] and in brain–heart interactions [117]. Non-linear approaches have been also largely exploited, based on using the auto-MI function to describe the complexity of the EEG signals, or the cross-MI function between different EEG channels to assess connectivity for sleepiness characterization [203].

Instead, the concept of MIR has been poorly investigated in brain-connectivity analysis. However, it has recently found applications in multivariate EEG, where interactions within specific frequency bands have been investigated to obtain markers of motor execution [204]. Non-linear estimators of MIR have been exploited to investigate the functional coupling between neural spike trains [195]. A method combining an information-theoretical approach based on the concept of MIR and statistical methods was exploited in [205] to infer connectivity in complex networks, such as the human brain, using time-series data.

Concepts of redundancy and synergy have been widely implemented in neuroscience in measures such as II and frameworks such as PID to study multiple interactions [206,207,208,209,210], e.g., in the context of brain–heart interactions [211], neural spike recordings [212], resting brains, and epileptic seizures [194,213,214]. Moreover, II was applied to human electrocorticography data in [185], revealing some aspects of multivariate brain interactions that withstand the epileptogenic zone, i.e., where the epileptic seizures are triggered. The epileptogenic structure was investigated in terms of the redundant/synergistic nature of these interactions.

A measure worth mentioning is the so-called information storage, also known as self-entropy (SE) [196]. Even though it is not a measure of brain connectivity, since it is associated with the analysis of single EEG channels, it has allowed researchers to map the spatial distribution of the complexity of brain dynamics, with reference to the amount of information in the past of a neural process that serves to predict part of the information contained in the future of that process [215]. Indeed, the lower the dynamical complexity in a given brain area (thus, higher regularity), the higher the information stored in that area. The measure of SE has been used especially in non-linear EEG analysis [196,215], but its parametric formulation in terms of AR model variances [216] has also been exploited to study scalp and source brain connectivity [200]. The feasibility of the linear approach was demonstrated in detecting modifications of the patterns of information storage in a group of children manifesting episodes of focal and generalized epilepsy [200].

### 4.4. Other Connectivity Estimators

Brain connectivity can be estimated through a large number of analyses applied to EEG data. Multivariate time-series analysis has traditionally relied on the use of linear methods in the time and frequency domains. Nevertheless, these methods are insufficient for capturing non-linear features in signals, especially in neurophysiological data where non-linearity is a characteristic of neuronal activity [108]. This has driven the exploration of alternative techniques that are not limited by this constraint [108,217]. Moreover, the utilization of AR models with constant parameters, and the underlying hypotheses of Gaussianity and WSS of the data, can be key limitations when stationarity is not verified [218]. A number of approaches have been developed to overcome this issue, providing time-varying extensions of linear model-based connectivity estimators using adaptive AR models with time-resolved parameters, in which the AR parameters are functions of time [174,219,220].

In this section, we discuss the main features of commonly used data-driven connectivity estimators which do not rely on data-generation models (e.g., phase-synchronization measures), and emerging trends in the field of brain connectivity (e.g., high-order interactions and complex networks tools).

#### 4.4.1. Phase Synchronization

Phase synchronization is a common phenomenon extensively studied in the literature [6,30,107,221,222,223], based on the evidence that the phases of two interacting signals can be synchronized, even if their amplitudes remain uncorrelated. Generally, measures of phase synchronization are frequency-specific. The complex-valued coherence is an example where the phase difference between the two signals at different frequencies can be interpreted as a time delay between the corresponding time series [6]. Specifically, the slope of the phase difference spectrum of the complex coherence (phase-lag) can be used to estimate the time delay between the two signals unambiguously, since the phase difference is observed over a range of frequencies and not in a single frequency bin [6]. In a noise-free environment, the slope of the phase is directly proportional to the delay between signals, a concept known as group delay in signal processing [224]. Furthermore, the sign and magnitude of phase-lag have been used to infer the directions of information transmission and delay [225]. Noteworthily, in the time domain, this corresponds to identifying the time lag of maximal correlation and its magnitude, which have been used to retrieve information flow between brain areas [226]. Ultimately, the cross-correlation function and the spectral complex-valued coherence have been utilized as measures of unidirectional directed neuronal interactions exerting their largest influences at a specific time delay *k*. Nevertheless, interpreting the cross-correlation function and the spectral coherence can be troublesome when dealing with complex, bidirectional interactions over multiple delays, which is common in most EEG cortical connections. Phase lag has been found to be near-zero in synchronous cortical networks, even anatomically distant between each other, which renders its use as a measure to identify directional interdependencies ineffective [149].

Other metrics of phase synchronization were introduced in the past years, among which are the phase slope index (PSI) [227] and the phase locking value (PLV) [107].

The PSI was proposed as a phase-synchronization measure derived from the complex coherence function [227], quantifying the change in the phase difference between consecutive bins. The weighted PSI stability, a variant of the PSI, has been defined as an artifact-resistant measure to detect cognitive EEG activity during locomotion [228].

The PLV was defined as the absolute value of the mean phase difference between the two signals, expressed as a complex unit-length vector [107]. PLV is thought to be an appropriate approach for quantifying phase synchronization between neural signals. Especially when dealing with EEG data, it does not require stationarity [107] and is robust to fluctuations in amplitude as opposed to coherence, which generally confounds the consistency of phase difference with amplitude correlation [6,107].

More recently, the imaginary part of the coherence was proposed to eliminate the impact of zero-lag correlations caused by volume conduction [229]. A comparison between methods which do not remove the zero-lag-phase connectivity and approaches which instead eliminate this effect were proposed in [38], including an application to magneto/electroencephalography source connectivity.

As regards applications to brain data, we refer the reader to [230,231,232,233,234,235] for further details on phase-coupling-based approaches reflecting various cognitive processes.

Moreover, it should be underlined that another coupling mode between neuronal oscillations does exist, being referred to as amplitude-coupling and reflecting the temporal co-modulation of the amplitude (or power) of neuronal oscillations. We refer the reader to [222,236,237] for further details on this kind of neuronal interactions.

#### 4.4.2. High-Order Interactions

The brain is a complex network of interacting neural populations, whose functional connections are investigated in the emerging field of network neuroscience [238,239]. Given the time series reflecting the dynamic activity of the network units, functional interdependencies are typically assessed by computing pairwise measures describing the interactions between two units only. However, despite their common use, research studies show that such measures do not accurately represent the connectivity structure of brain networks, since the obtained patterns can be blurred by the large number of spurious connections due to unobserved variables [112,118]. Multivariate approaches based on conditioning or partialization procedures have been exploited to remove the effects of third inputs on the observed patterns of connectivity [16,113]. Indeed, it has been demonstrated that higher-order interactions (HOIs), i.e., interactions involving three or more units, greatly impact the overall behavior of brain networks [213]. In recent years, various information-theory-based metrics have been proposed to measure HOIs among multiple time series, which aim to identify redundant or synergistic information shared by groups of random variables or processes [187,189,213,216,240]. Interaction information (15) is an example, but its computation involves only three variables or groups of variables.

A recently proposed measure, the so-called O-information (OI), has been used to investigate synergy- and redundancy-dominated interactions in networks of multiple interacting units [25]. Its symmetric nature, scalability with the network size, and the possibility to compute it for dynamic processes were shown [26]. To complement the global assessment provided by the OI, a new measure has been proposed reflecting the gradients of the OI as low-order descriptors (i.e., univariate and pairwise) that can characterize how high-order effects are localized across a system of interest [28]. This measure has been successfully applied in the context of econometrics, showing the potential impact it could also have on the study of physiological systems such as the human brain [28].

Moreover, a new framework was introduced for the time- and frequency-domain analysis of HOIs in multivariate stochastic processes mapping the activity of network systems [27]. The new measure of O-information rate (OIR) was defined which generalizes the MIR of bivariate processes using the same rationale, whereby the OI generalizes the MI between random variables. In the context of the VAR formulation of multivariate Gaussian stochastic processes [21], a causal decomposition and a spectral expansion of the OIR was provided, thereby connecting it with well-known and widely used measures of coupling and GC formulated in the time and frequency domains [12,24].

This concept of multivariate analysis is becoming more and more popular for describing the activity and the interactions among subunits forming a system. The analysis of multiple time series interacting in complex manners and representing the dynamic activity of brain networks, together with the tools of information theory described above, is an emerging trend that should be taken into account when dealing with brain data [27,241].

#### 4.4.3. Complex Network Measures

Graph theory is a useful tool with which to model the structural and functional connections between nodes in complex brain networks [29,30,31]. Nodes represent brain regions; and edges represent synapses, pathways, or statistical dependencies between neural units. The nature of nodes and edges varies depending on the brain mapping method, anatomical parcellation scheme, and measure of connectivity used [242]. The choice of a specific combination of these largely determines the interpretation of network topology [243].

Ideally, nodes in a network should correspond to brain regions that exhibit anatomical or functional connections, and connections are differentiated based on several factors, including the type of connectivity (anatomical, functional, or effective), their weight (binary or weighted graphs) and direction (undirected or directed graphs). Weighted directed variants are typically generalizations of binary undirected variants [239]. Binary and weighted graphs differ in their representations of connections. Binary graphs indicate only the presence or absence of a connection, and weighted graphs provide information about the strength of that connection [239]. Weighted graphs can exclude non-significant links, which are generally discarded by applying arbitrary weight thresholds [244,245,246]. Undirected and directed graphs are represented by symmetric and asymmetric adjacency matrices, respectively. Symmetric measures are used to examine the degree of association in undirected graphs, and asymmetrical metrics provide information on the direction of the connections in directed graphs also [239,247].

The structural and functional properties of graphs of brain networks can be quantitatively investigated through measures of [239]:

- Functional segregation, e.g., clustering coefficients [248,249,250,251,252] and modularity [253].
- Functional integration, based on the concept of path [248,254] and estimating the ease of communication between brain areas. These measures have been found useful in studies related to obsessive-compulsive disorders, since their alterations seem to be correlated with the severity of the illness [255,256]. Networks which are simultaneously highly segregated and integrated are referred to as small-world networks; a measure of small-worldness was proposed to describe this property [248,257].
- Network motifs, which are subgraphs showing patterns of local connectivity.
- Node and edge centrality, based on the idea that specific combinations of nodes and links can control information flow [249,258,259,260,261].
- Network resilience, based on the evidence that anatomical connectivity influences the ability of neuropathological lesions to affect brain activity.

Many of the topological indices which characterize a network have been implemented in freely available software packages, such as Brain Connectivity Toolbox [239] and EEG-NET [262] in Matlab, or NetworkX [263] in Python.

The statistical evaluation of these network statistics requires the design of appropriate null-hypothesis networks, involving the choice of random or ordered graph models which preserve basic features of the original network [264].

Ultimately, complex network tools are particularly important in understanding connectivity, as they provide a mathematical framework for modeling and analyzing the network of connections between brain regions, and to better understand brain function and disorders, such as Alzheimer’s disease, autism, and schizophrenia, and their impacts on brain connectivity over time, which can help to track the progression of brain diseases and inform the development of new treatments [30,261,265,266,267,268,269].

### 4.5. Statistical Validation Approaches

It is common practice to statistically validate the estimated metrics of connectivity, which correspond to assessing whether the two investigated (blocks of) time series are significantly coupled, i.e., whether the estimated value of connectivity is significantly nonzero. Indeed, due to practical estimation problems, nonzero values of the estimated connectivity index can occur even in the absence of a real coupling between the two considered series. To face this issue, the statistical significance of a given measure is typically assessed by estimating its distribution and comparing it to a given arbitrary threshold. However, a rigorous and more powerful method would consist in defining a threshold level on the basis of statistical criteria derived from the sampling (theoretical or empirical) distribution of the used estimator. Theoretical approaches have been used to assess the statistical significance of the Coh [270], PDC [172,271], DTF [165], and DC [167] estimators [272], but they present some limitations which cannot be neglected in real applications [121]. Therefore, the empirical distribution of the considered index for the estimation of a threshold level has been used in place of theoretical approaches [273]. The empirical distribution is commonly obtained by exploiting the method of surrogate data, a technique originally proposed to investigate the existence of non-linear dynamics in time series [274,275] but later exploited to test the significance of coupling measures in EEG recordings [107]. According to this approach, the index is computed over a set of surrogate time series, which are derived from the original series by a procedure mimicking their properties but removing their coupling. The confidence interval (CI) of the empirical distribution is then computed under the null hypothesis of full uncoupling between the time series; the 100(1−α)*th* percentile of the distribution (which represent the threshold value) is then compared with the observed value, and the null hypothesis is accepted or rejected at the α significance level depending on the position of the observed value with respect to the threshold [276]. Indeed, if the index assessed over the original series is above the threshold, the null hypothesis is rejected with type I error probability below α.

Different algorithms have been proposed to generate surrogate time series sharing some given properties with the original but being uncoupled:

- Randomly shuffled surrogates [277], which are realizations of i.i.d. stochastic processes with the same mean, variance, and probability distribution as the original series, generated by randomly permuting in temporal order the samples of the original series; this procedure destroys the autocorrelation function.
- Fourier transform (FT) or phase-randomized surrogates [274], which are realizations of linear stochastic processes with the same power spectra as the original series, obtained by a phase randomization procedure applied independently to each series.
- Iterative amplitude adjusted FT (iAAFT) surrogates [275], which are realizations of linear stochastic processes with the same autocorrelations and probability distributions as the original series, and the power spectra are the best approximations of the original ones according to the number of iterations.
- AR surrogates [13], which are realizations of linear stochastic processes with the same power spectra as the original series, constructed by fitting an AR model to each of the original series, using independent white noises as model inputs.

Surrogates preserving the power spectrum of the original series (FT, iAAFT, AR) are recommended to avoid false coupling detections in the presence of oscillations occurring at nearby frequencies but due to different mechanisms, as may frequently happen with brain oscillations [276].

Moreover, it should be highlighted that the null hypothesis of full uncoupling is often used in directionality analysis, since it is compatible with the absence of a causal relation [168,278,279,280,281,282,283], but it may overestimate the detection of causality, as it assumes the complete lack of interaction, thereby also neglecting alternative dependencies. Surrogates that align with the null hypothesis of no causal interaction in the direction of interest should be generated. To this end, an approach based on a modified FT algorithm has been proposed and applied to multichannel EEG signals [160], consisting of the selective destruction of causality only over the investigated direction of interaction, while leaving untouched causal effects over alternative pathways.

In brain connectivity studies, surrogate data must be consistent with the null hypothesis of no neural interaction while sharing all other properties of the original data. Common approaches include the use of randomly shuffled surrogates [168,284,285,286,287] or phase-randomized signals [204,288,289]. However, these methods remove all dependencies between time series, thereby not accounting that EEG data, and their source estimates show significant correlations even under the null hypothesis of independent sources [290,291]. This issue is currently debated in the literature. Many research studies have focused on the generation of surrogate data with a realistic correlation structure [291,292,293,294,295].

## 5. EEG Acquisition and Pre-Processing

**Figure 3:**
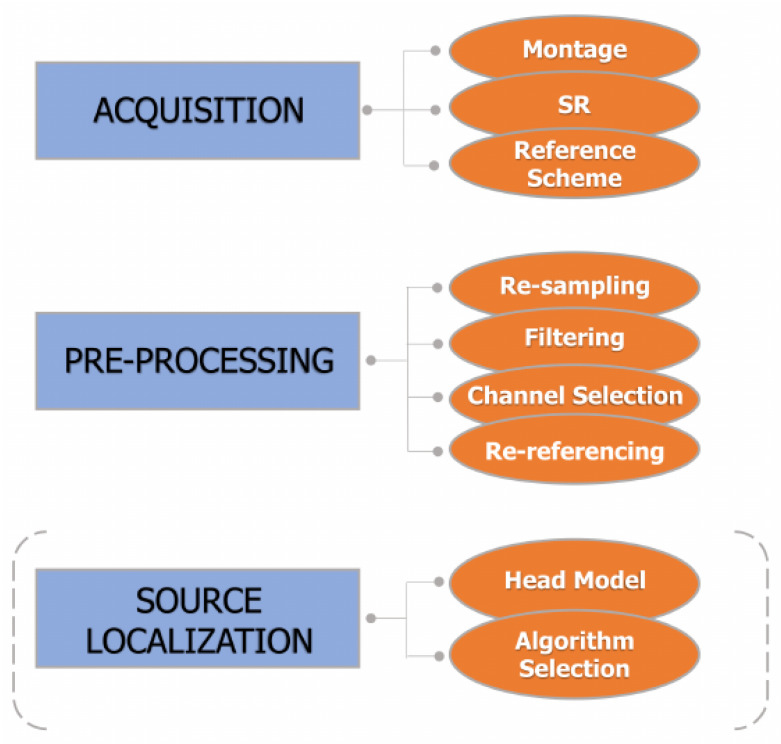
Main steps in EEG acquisition and pre-processing. In general, source localization is not mandatory, as represented by the dashed round brackets.

**Figure 4:**
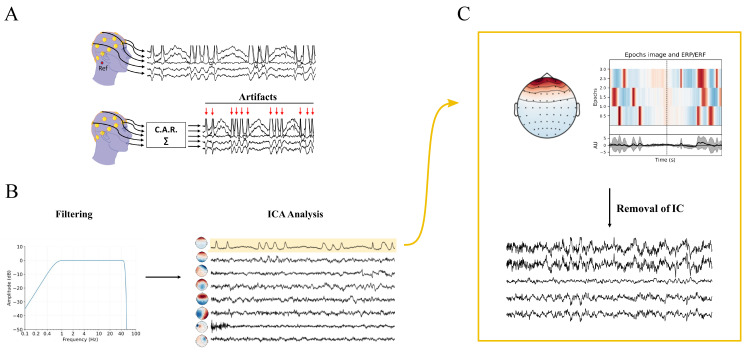
Schematic representation of the pre-processing pipeline applied to EEG signals acquired on the scalp (s253 recording of the subject 2 that could be found in https://eeglab.org/tutorials/10_Group_analysis/study_creation.html#description-of-the-5-subject-experiment-tutorial-data, accessed on 15 February 2023). (**A**) Unipolar EEG signals are acquired using a mastoid reference (Ref, in red). For clarity, only a limited number of the recorded signals, among the original 30 channels, is plotted. The average re-referencing process and the pre-processed signals are illustrated below. Notably, red arrows indicate blinking artifacts that are clearly visible. (**B**) The re-referenced signals are filtered using a 1–45 Hz zero-phase pass-band filter, followed by independent component analysis (ICA) to extract eight independent components (ICs), shown on the right. (**C**) The first IC, suspected to be an artifact, is analyzed, with a scalp-shaped heatmap assessing its localization in the frontal area and its temporary coincidence with the artifacts shown in panel (**A**). After removing the first IC, the cleaned signal is plotted at the bottom of the panel.

The acquisition and conditioning of the EEG signal represent two important aspects with effects on the entire subsequent processing chain. The main steps of acquisition and pre-processing are indicated in Figure 3. An example of application of the pre-processing pipeline to experimental EEG is shown in Figure 4. Here, we introduce some basic notions and techniques, focusing on the effects on FC estimation.

Sampling frequency, the number of electrodes, and their positioning, each have an important role in assessing connectivity; too low of a sampling frequency cannot be used to analyze high-frequency bands for the Nyquist–Shannon sampling theorem [296], and the electrode density defines with which accuracy and reliability further processing will be performed [40].

An EEG signal is a temporal sequence of electric potential values. The potential is measured with respect to a reference, which should be ideally the potential at a point at infinity (thus, with a stable zero value). In practice, an ideal reference cannot be found, and any specific choice will affect the evaluated connectivity [6,57]. Unfortunately, as for most of the FC analysis pipeline, there is no gold standard for referencing, and this is clearly a problem for cross-study comparability [297]. An ideal reference point needs to be neutral with respect to the measured signal, and many reference points have been proposed and tested. The most common ones are applied in a specific part of the human body, such as linked mastoids/ears (LM) and left mastoid references (LR); or channel-based, such as (usually) the central electrode Cz.

Since reference montages are inevitably influenced by brain activity (as said, there is no neutral reference), recordings are generally digitally re-referenced offline with respect to a more neutral reference (this aspect is discussed in the next section).

Although at the very beginning of EEG studies, bipolar recording was used [298], nowadays, the vast majority of works based on EEG connectivity is performed using unipolar recording. Then, a bipolar recording is obtained as the subtraction of the signals of two adjacent electrodes. This is mathematically an approximation of a first-order spatial derivative:

(22) $$c\approx v_{r}^{\left(n+1\right)}-v_{r}^{\left(n\right)}\propto \frac{\Delta v_{r}}{d}$$

where c is the bipolar recording between electrode *n* and n+1, $v_{r}^{\left(n\right)}$ is the potential at the electrode *n*, and *d* is the distance between the two electrodes. This type of recordings acts as a high-pass filter, making it more affected by random noise and less sensitive to signals from deep neural sources but more stable to common-mode interference and selective of focal activities [299]. For this reason, bipolar montage is still used in clinical applications, especially in epilepsy [300].

In every EEG processing pipeline, a fundamental role is covered by a consistent pre-processing phase. That is due to the properties of the EEG signal, which is generally characterized by a fairly low signal-to-noise ratio (SNR), compared to other bioelectrical signals such as ECG or EMG, and a low spatial resolution (balanced by a high temporal resolution). Pre-processing is aimed at increasing the SNR, but it could also affect the properties of the neural contributions to EEG, which are of interest.

In the following, some considerations are given on the effects of the most common pre-processing methods in terms of the possible distortions, disruptions, or alterations in the connectivity information content present in the signals.

### 5.1. Resampling

Nowadays, EEG signal is usually acquired with a sampling rate (SR) equal or superior to 128 Hz, since this value is the lower base-2-power that permits one to capture most of the information content in EEG signal and a big part of the γ band. It is noticeable that high-frequency oscillations (HFOs), such as ripples (80–200 Hz) and fast ripples (200–500 Hz), will not be captured with these SRs [51,52,53,54]. Moreover, there are studies suggesting that low SRs affect correlation dimensions, and in general, non-linear metrics [55,56].

However, some FC studies are focused on lower frequency bands, suggesting that a procedure of downsampling would be beneficial because it will lighten the signal, making further processing faster and digital filter design simpler. For this reason, signal resampling should be the first pre-processing step, if needed. Care must be taken in avoiding aliasing, by applying a proper low-pass antialiasing filter, which cuts-off the higher frequencies not representable with the new SR. Moreover, high SRs result in an increased successive sample correlation that generates spurious patterns in the signal. This phenomenon affects all the methods based on time-delay embedding, as they rely on the assumption that the signal is uniformly sampled.

### 5.2. Filtering and Artifact Rejection

Filtering EEG is a necessary step for the FC analysis pipeline, not only to extract the principal EEG frequency waves, but particularly to reduce the amounts of noise and artifacts present in the signal and to enhance its SNR. Research suggests the use of finite impulse response (FIR) causal filters that could be used also for real-time (RT) applications, or infinite impulse response (IIR) filters, which are less demanding in terms of filter orders but distort phase, unless they are applied with reverse filtering, thereby making the process non-causal and not applicable for RT applications. In general, if sharp cut-offs are not needed for the analysis, FIR filters are recommended, since they are always stable and easier to control [301].

If one is interested in investigating FC in the γ band, electrical line noise at 50 or 60 Hz could be a problem, since it is not fully removable with a low-pass filter. Notch filters are basically band-stop filters with a very narrow transition phase in the frequency domain, which in turn leads to an inevitable distortion of the signal in the time domain, such as smearing artifacts [301]. To avoid this problem, some alternatives have been developed. A discrete Fourier transform (DFT) filter is obtained by subtracting from the signal an estimation of the interference obtained by fitting the signal with a combination of sine and cosine with the same frequency as the interference. It avoids potential distortions of components external to the power-line frequency. It assumes that the power-line noise has constant power in the analyzed signal segment. As this hypothesis is not strictly verified in practice, it is recommended to apply the DFT technique to short data segments (1 s or less).

Another proposed technique is CleanLine, a regression-based method that makes use of a sliding window and multitapers to transform data from the time domain to the frequency domain, thereby estimating the characteristics of the power line noise signal with a regression model and subtracting it from the data [302]. This method eliminates only the deterministic line components, which are optimal, since EEG signal is a stochastic process, but in the presence of strong non-stationary artifacts, it may fail [303].

Alternatively, there is the possibility of reducing power-line noise through spectral interpolation [304], which makes use of DFT to transform temporal data in the frequency domain, identify and remove the line noise component in the magnitude spectrum, and convert it back into the time domain through inverse discrete Fourier transform (iDFT).

It is pretty normal that EEG signals can be corrupted by many types of artifacts, defined as any undesired signal which affects the EEG signal whose origin cannot be identified in neural activity. Generators of these undesirable signals could be physiological, such as ocular artifacts (OAs such as eye blink, saccade movement, rapid eye movements), head or facial movements, muscle contraction, or cardiac activity [305,306]. Power-line noise, electrode movements (due to non-properly connected electrodes), and interference with other electrical instrumentation are non-physiological artifacts [307]. Artifact management is crucial in the analysis of connectivity. In fact, their presence in multiple electrodes can result in overestimation of brain connectivity, skewing the results [308,309].

Luckily, in many cases, groups of artifacts could be described by some characteristics that can be exploited to recognize them [310], even if it is known that artifacts can mimic every physiological pattern in both quantitative and qualitative analysis [311].

The most effective way for dealing with artifacts is to apply prevention in the phase of acquisition to avoid as much as possible their presence, for example, by making acquisitions in a controlled environment, double-checking the correct positioning of electrodes, or instructing the patients to avoid blinking the eyes in certain moments of the acquisition. In fact, eye blinks are by far the most common source of artifacts in the EEG signal, especially in the frontal electrodes [312,313]. They are shown as rapid, high-amplitude waveforms present in many channels, whose magnitudes exponentially decrease from frontal to occipital electrodes. However, saccade movements produce artifacts, generating an electrooculographic signal (EOG). Acquisition of this signal, concurrently with the EEG signal, is known to be a great advantage in identifying and removing ocular artifacts, since vertical (VEOG), horizontal (HEOG), and radial (REOG) signals diffuse in different ways through the scalp [314]. With the hypothesis that the actual EEG signal could be obtained as a linear combination of the artifact-free EEG signal and the recorded EOG, a regression model can be built to identify the interference of the EOG in the EEG signal, and thus, subtracting it [315].

Once the parts of the signals corrupted by the artifacts have been identified, it is still common practice to eliminate these portions, avoiding alterations on the signal that could lead to spurious connectivity. As for channel rejection, however, it is preferable to retain as much information as possible [316].

Unfortunately, it often happens that only the EEG signal is available, without any other knowledge of the artifacts corrupting it. In such cases, blind source separation (BSS) techniques are utilized to identify artifacts. BSS methods usually consider the EEG as a linear mixture of uncorrelated or independent sources, either neural or artifactual. Given X, the M×n EEG matrix of *M* channels (the sensors) and *n* samples; A, the M×k unknown mixing matrix; S, the k×n matrix of unknown *k* sources; and V, the M×n noise matrix, the model could be written as:

(23) X=AS+V.

As for all the inverse problems, this is an ill-posed model, since there are infinite combinations of sources that could produce the same EEG matrix; thus, further hypotheses need to be formulated to converge to a unique solution.

Independent component analysis (ICA) is a widely used method introduced in the EEG by [317], which allows one to identify and isolate the sources that generate the recorded signal. Although there are more than 15 ICA-based algorithms [318,319], only a few are the currently most used for artifact rejection in biomedical signals, which are FastICA [320,321], SOBI [322], and Infomax [318]. As its name suggests, ICA’s (strong) fundamental hypothesis is that the sources should be statistically independent of each other, and they are called independent components. Thus, ICA can be considered as an extension of principal component analysis (PCA), which assumes sources to be only uncorrelated [323]. Moreover, it is noticeable that processed signals with ICA methods are assumed to be stationary, meaning that their frequency power spectrum should not change over time. For real problems, these assumptions could not be truly satisfied; nevertheless, ICA-based algorithms have been continuously reported to be effective at detecting and removing artifacts in EEG [310]. The reasons behind their effectiveness are searchable due to two important facts. The first is that the artifacts are in general sufficiently independent to not interfere with the first fundamental assumption; the second is that even if the EEG signal is not stationary, it can be processed as a subdivision of relatively small time windows that make it WSS. Some authors suggest that 10 seconds could be used as a reasonable time window [324]. In any case, it is also not suggested to use very short signal segments, since ICA algorithms make use of statistical analysis, and this could affect their reliability and performances [324].

Connectivity measures are generally affected by both the artifact (and noise) present in the signal and the techniques used to reduce it. There are studies showing how GC is theoretically invariant with filtering [153], but it has been shown successively that filtering and downsampling actually have important effects in practical applications [91,303,325]. An improved and modified version of the phase lag index, the weighted phase lag index [326], was developed as a noise and artifact-resistant connectivity metric. In a subsequent study, it was used to assess connectivity by acquiring the EEG signal while the subject was walking, obtaining comparable results recovered while standing [228]. However, it is important to point out that this study was performed using high-density EEG recording, and to our knowledge, there are no studies that could confirm this resilience for lower density EEG systems.

ICA could generate non-linear and non-stationary phase distortion on an EEG signal, as demonstrated with simulation studies [327]. Indeed, it is recommended to filter only when strictly necessary and with caution [328].

### 5.3. Bad Channel Identification, Rejection, and Interpolation

It could happen that some EEG channels present a high number of artifacts (eye blink, muscular noise, etc.) or noise, due to bad electrode-scalp contact. In these cases, the rejection of these channels could be an option. However, it is necessary to check whether the deleted channels are not fundamental and the remaining channels are sufficient to carry on the analysis, considering also that deleting channels will result in an important loss of information that is likely not recovered anymore. Some authors discussed the criteria for detection of bad channels and suggested considering the proportion of bad channels with respect to the total to assess the quality of the dataset (for example, imposing a maximum of 5%) [329].

Identification of bad channels could be performed visually or automatically. Visual inspection requires certain experience with EEG signals to decide if a channel is actually not recoverable and needs to be rejected [330,331]. Indeed, this process is highly subjective. Automatic detection of bad channels could be performed in various ways. A channel correlation method identifies the bad channels by comparing their divergence from the Pearson correlation distribution among each pair of channels with the other couples. This method assumes high correlations between channels due to the volume-conduction effect proper of the EEG signal, which is discussed in detail in the source localization section. High standard deviation of an EEG signal can be an indicator of the presence of a great amount of noise. By setting a proper threshold, the standard deviation can be a useful index for identifying bad channels.

Once the bad channels have been located, two options are available: their removal from the dataset or their replacement with dummy channels by interpolation of the retained ones. Due to the physiological curvature of the scalp, spherical spline interpolation [332] is often used.

### 5.4. Re-Referencing

As described in the previous section, many decisions are made during the acquisition of the signals; however, some of them are revertible. Re-referencing and down-sampling is one of them. It consists of changing the (common) reference of each EEG channel into another one by performing for each channel the addition of a fixed value (notice that it is a fixed value in space, i.e., common for all channels, but not in general constant in time).

Re-reference with respect to a new reference channel can be performed by simply subtracting each channel with the new reference. In most cases, average reference (CAR) is considered to be a good choice, especially if electrodes cover a large portion of the scalp with the assumption that the algebraic sum of currents must be zero in the case of uniform density of sources [333]. It consists of referencing all the electrode signals with respect to a virtual reference that is the arithmetic mean of all the channel signals. Due to charge conservation, the integral of the potential over a closed surface surrounding the neural sources is zero. Obviously, the EEG channels give only a discrete sampling of a portion of that surface, so that the virtual reference can be assumed to be only approximately zero. However, it could be much better than using a single location as reference, such as an EEG channel or the mastoid (both LM and LR), which have been shown to generate larger distortion than the average reference [308].

It is evident that the hypothesis of the average reference becomes increasingly inadequate for low-density EEG recordings [334]. Furthermore, noisy EEG channels pose a challenge, as they can significantly alter the average potential estimation. To overcome this problem, other referencing methods have been proposed, such as the surface Laplacian, which represent a true reference-free transformation [335], which has been used in many contexts [61]. Another type of referencing to limit interferences is known as the reference electrode standardization technique (REST) [336]. It consists of a linear transformation of the EEG matrix signal into an approximately zero-reference one. This method has been validated by many simulation studies [337,338,339], and it was suggested that it could be the best choice for most cognitive studies and clinical EEG problems [299,340,341,342]. It is particularly advocated if a sufficient number of electrodes is present (≥10) [297,299,337,341].

Improvement of the CAR technique by adding a robust maximum likelihood estimator was proposed in [343]: this method was compared with REST, and it seems to give better performance.

The scalp power spectra map is demonstrated to be systematically altered in spontaneous EEG with a non-neutral reference signal mixed in other channels, and it is heavily dependent on the chosen reference scheme [344]. Additionally, the choice of reference can significantly impact measures of correlation in both time and frequency domains [345] by introducing zero-lag correlations, making the interpretation of EEG functional brain-network characteristics more challenging. In general, it is demonstrated that approximations of the infinity reference (like REST) have better performance with respect to other referencing schemes in estimating FC, especially when using coherence [346].

## 6. Source Connectivity Analysis

Measuring information dynamics from EEG signals on the scalp is not straightforward due to the impact of volume conduction, which can modify or obscure the patterns of information flow across EEG electrodes [196]. This effect is due to the electrical conductance of the skull, which serves as a support to diffuse neural activity in all the directions and for this reason is also known as field spread problem. The neural current sources are related to the Poisson equation to the electrical potential, which diffuses across the scalp and can be measured by many electrodes, also pretty far from the original source. This is the reason why interpreting scalp-level connectivity requires caution. In fact, the estimated FC between two electrodes could be reflecting the activation of a single brain region, rather than two functionally connected regions. Even though this effect can be compensated for when working with scalp EEG signals [196,197], it is often recommended to use source signal reconstruction to obtain a more accurate representation of the underlying neural network. This is because the source-based network representation is considered a more accurate approximation of the real neural network structure [347].

The general pipeline that source localization algorithms follow is based on this two-step loop [348]:

Forward problem—definition of a set of sources and their characteristics and simulation of the signal that would be measured (i.e., the potential on the scalp) knowing the physical characteristics of the medium that makes it diffuse;
Inverse problem—comparison of the signal generated by the head model with the actual measured EEG and adjustment of the parameters of the source model to make them as similar as possible.

The first part, also known as estimation problem, can be carried out by defining a proper head model using boundary element models (BEMs) or finite element models (FEM). The second part of the pipeline is also called the appraisal problem, and it is not trivial at all, being one of the fundamental challenges in EEG processing analysis. This process is in fact formally an inverse ill-posed problem, where there exists an infinite number of combinations of sources that can explain the acquired signal [349].

Both these fundamental parts of the source localization problem are detailed in the next paragraphs.

### 6.1. Forward Problem and Head Models

In the past, the first attempts of EEG source localization were based on the strong a priori assumptions that only one source is active at a certain time instant and that the head can be approximated as a sphere which is homogeneously conductive. These hypotheses allowed scientists to find an analytical solution for the forward problem; this is computationally convenient, but a non-realistic head model was used. Nowadays, it is not recommended to use whole homogeneous spherical head models, but possibly to make use of spherical multi-shell models. They divide the head into a certain number of concentric spheres (generally 3 or 4) where each layer can be defined as homogeneously conductive and isotropic. This is a more realistic approach, since the skull and brain, for example, have consistently different conductivity values (with a ratio estimated to be around 1:20 [350]).

Probably the most popular head models are the BEMs [351], which were introduced to incorporate even more anatomically and physiologically relevant information. In these models, the surface boundaries of each tissue of the head—separating volumes considered as isotropic and homogeneous—are sampled by a mesh of triangles. This relatively coarse space discretization allows a more accurate definition of the head (generally, the number of triangles involved is a few thousand), and consequently, of the conductance present in different regions with respect to the multi-shell spherical head models.

When the space discretization is finer and applied to the whole 3-dimensional space, a FEM is used [352]. Each elemental volume is subdivided by points disposed in a dense grid—up to one hundred-thousands points—which allows a very precise definition of the heads’ volumes and permits their anisotropy in the conductivity. Even if this approach is by far the most accurate, it is still difficult to perform due to the extremely high computational power needed to fulfill the calculations.

### 6.2. Inverse Problem

In order to obtain a unique solution of the inverse problem, it is necessary to impose some assumptions on the sources that originate the acquired signal. As presented in the previous paragraph, the choice of the head model is fundamental to representing the biophysical characteristics of the tissues in which the sources are immersed, and thus to compute accurately how the potentials are conveyed up to the scalp. The simplest way to define the sources is to consider them as current dipoles and solve an equivalent current dipole (ECD) problem [348]. Each of these dipoles has three degrees of freedom in space and a magnitude value (the so-called moment parameter). It is known that in not all the regions of the brain is it possible to find neural activity measurable in the scalp; thus, some space constraints are applied, whose accuracy is modulated by the head model.

Historically, the first approaches to solving the EEG inverse problem considered only few sources as generators of the scalp potential signal, limited by the number of the electrodes and distributed in the head [353]. Nonetheless, an underestimated number of sources will lead to a biased source localization generated by the missing dipoles in the model: thus, it is not recommendable for a connectivity analysis study. Nowadays, it is preferable to consider a large number of current dipoles (5000 or more), generally placed in fixed positions inside the gray matter [5], solving the so-called linear distributed dipole (LDD) inverse problem.

The most general algorithm for solving LDD is the minimum norm (MN) [354,355] that iteratively searches for the dipole distribution with minimum energy, allowing one to represent the recorded EEG. Despite it providing good results in terms of resolution and current estimation, it fails to address the issue of deep source localization in the outermost cortex [348]. To overcome this limitation, other algorithms based on MN have been designed. Low-resolution electromagnetic tomography (LORETA)-based algorithms [356] are probably the most utilized to solve the inverse problem in EEG. The original algorithm is based on an additional constraint of simultaneous and synchronous activation of the neighboring sources, which corresponds to the minimization of their Laplacian. However, this hypothesis generates a solution that has increased time resolution but decreased spatial resolution. Improvements in the original algorithm have been proposed, named sLORETA [357] and eLORETA [358]. The former assumes a standardization of the current density, resulting in a zero error localization error, and it has much better performances than MN method according to simulation studies with and without noise [348,359]. The latter is a more recent variant of the LORETA algorithm that focuses on estimating source localization, giving more importance to the deeper sources, maintaining a reduced localization error. As for the sLORETA, this algorithm is standardized and have a low spatial resolution, which could result in blurred images when space is subjected to regularization [348,358].

As previously said, connectivity measures are in general affected by the volume-conduction effect. However, it is worthwhile mentioning that some metrics are supposed to be more resilient than others, in particular, those based on phase differences, such as partial directed coherence [16] and the directed transfer function [18]. This is due to the fact that volume conduction is a zero-phase propagation, and thus, no phase difference is generated between channels [360]. Subsequent researchers have demonstrated with simulation studies that mixing of cortical source activities could indeed affect DTF [64,361]. However, it was successively pointed out that, in practice, this influence does not significantly distort the estimates and that correct results can be reached by applying an appropriate threshold to the propagation values obtained [362].

For a similar reason, the imaginary part of coherence is another connectivity metric that is theoretically resistant to artifacts generated by volume conduction [229]. In fact, if we consider the field spread effect as instantaneous, by discarding the real part of the coherence, all the potentially artifactual zero-lag interactions generated by volume-conduction effect will be removed. However, this approach carries a double cost. At first, eventual true zero-phase interact will not be detected, since they are all discarded with the rejection of the real part of coherence. Additionally, the imaginary component of coherence makes it difficult to determine the temporal order of processes, as they have their own periodicity [62].

The same discussions could be had also for other metrics based on phase difference, such as the phase slope index [30].

Thus, the most popular and effective method for FC estimation is to reconstruct the sources that could have generated the EEG signal and work directly with the estimated sources, which are not affected by the volume-conduction effect [363].

## 7. Discussion and Conclusions

In this review, we aimed to provide a comprehensive guide to data-driven FC analysis of EEG signals.

First, we briefly discussed the key concepts of structural, functional, and effective connectivity, with a specific focus on how these notions are connected to each other. From this, we specified the direction of the work, which is the investigation of FC intended as the presence of statistical dependencies between the recorded signals, performed through data-driven directed and non-directed approaches. Accordingly, we described the most-used metrics of connectivity in the time, frequency, and information domains, with a distinction between pairwise and multivariate approaches. This discussion was focused on AR linear modeling of the investigated time series, which implies linearity and Gaussianity of the data. Inherently model-free, information-theoretic measures were introduced in terms of their linear AR model-based interpretation, which allowed us to derive their frequency-specific counterparts with specific physiological meanings. Other connectivity estimators widely used in brain-connectivity analysis were also discussed, i.e., phase-synchronization measures, along with emerging trends, such as the framework of high-order interactions. The latter, though being still not very popular in EEG connectivity, is becoming very relevant for the study of network systems, and thus holds the potential to spread significantly in the field of brain connectivity. Nonetheless, complex network analysis, grounded on the notions of graph theory, was herein briefly discussed, as it is becoming a widely exploited tool to investigate the interdependencies and structures of complex brain networks.

In addition, we emphasized the importance of proper acquisition and pre-processing of the acquired EEG signals. These steps are often overlooked, but they are crucial for obtaining reliable and high-quality results. Among others, proper re-referencing and SNR enhancement are essential steps in EEG signal analysis that require ongoing updates to the latest techniques in order to be applied correctly.

Electroencephalography represents a highly valuable tool for estimating brain connectivity, due to its low-cost and non-invasive nature, and due to its good temporal resolution. However, a large number of challenges must still be addressed, such as the high quantity of acquisition artifacts and the volume conduction problem. Researchers should have a comprehensive understanding of these issues, as they can often play a crucial role in the selection of the appropriate metrics for evaluating FC. The literature regularly demonstrates that some of these metrics are more resilient to the distortions and corruptions caused by the aforementioned concerns.

It should be highlighted that some well-known problems arising during brain-connectivity analysis have not been discussed for brevity, such as the issues of sample bias and computational load. It may be also crucial to delve into these potential pitfalls, as the former can significantly impact the evaluation of connectivity estimates if not properly addressed and mitigated, and the latter limits real-time applications, where it is necessary for the pre-processing and connectivity estimation to occur almost immediately.

It is clear that the field of connectivity estimation from EEG signals is constantly advancing and improving. New and innovative approaches are being developed every year. By proposing some of them in the present review, we encourage the readers to approach the problem of inferring functional brain connectivity from different and newborn perspectives, in order to keep up with the ongoing fascination and excitement that surround this challenging and complex area of research. The future looks bright for the development of even more sophisticated and effective connectivity estimation methods, which will no doubt play a significant role in our understanding of the brain and its functions.

## Data Availability

The signal shown in Figure 4 is file syn02-s253-clean.set from https://eeglab.org/tutorials/10_Group_analysis/study_creation.html#description-of-the-5-subject-experiment-tutorial-data, accessed on 15 February 2023.

## Abbreviations

The following abbreviations are used in this manuscript:

AR	Autoregressive
bCoh	Block Coherence
bDC	Block Directed Coherence
BEM	Boundary Element Models
bPDC	Block Partial Directed Coherence
BSS	Blind Source Separation
CAR	Average Reference
cGC	Conditional Granger Causality
cIC	Conditional Instantaneous Causality
cMI	Conditional Mutual Information
Coh	Coherence
Corr	Correlation
cTD	Conditional Total Dependence
cTE	Conditional Transfer Entropy
DC	Directed Coherence
DFT	Discrete Fourier Transform
DTF	Directed Transfer Function
EC	Effective Connectivity
ECD	Equivalent Current Dipole
ECoG	Electrocorticography
EEG	Electroencephalogram
EOG	Electrooculogram
FC	Functional Connectivity
FEM	Finite Element Models
FIR	Finite Impulse Response
fMRI	Functional Magnetic Resonance Imaging
GC	Granger Causality
HOIs	High-Order Interactions
IC	Instantaneous Causality
ICA	Independent Component Analysis
II	Interaction Information
i.i.d	Independent Identically Distributed
IIR	Infinite Impulse Response
i.d	Identically Distributed
ITE	Interaction Transfer Entropy
jTD	Joint Total Dependence
jTE	Joint Transfer Entropy
LDD	Linear Distributed Dipole
MI	Mutual Information
MIR	Mutual Information Rate
PCA	Principal Component Analysis
PCC	Pearson Correlation Coefficient
PCoh	Partial Coherence
PCorr	Partial Correlation
PDC	Partial Directed Coherence
PLV	Phase Locking Value
PRho	Partial Correlation Coefficient
PSD	Power Spectral Density
PSI	Phase Slope Index
R	Redundancy
REST	Reference Electrode Standardization Technique
Rho	Correlation Coefficient
S	Synergy
SC	Structural Connectivity
SNR	Signal-to-Noise Ratio
SR	Sampling Rate
TD	Total Dependence
TE	Transfer Entropy
VAR	Vector Autoregressive
WSS	Wide-Sense Stationary
